# Eco‐Friendly Synthesis of Cobalt Phthalocyanine for High‐Efficiency ${\mathrm{CO}}_{2}$ Electroreduction

**DOI:** 10.1002/chem.202502264

**Published:** 2025-11-26

**Authors:** Gloria Zanotti, Federica Palmeri, Venanzio Raglione, Abdolhamid Khodadadi, Camille Tabaries, Jerome Capitolis, Mathieu Prevot, Laurent Piccolo, Mahmoud Zendehdel, Roberto Flammini, Giorgio Contini

**Affiliations:** ^1^ Istituto di Struttura della Materia‐CNR (ISM‐CNR) Montelibretti Italy; ^2^ Department of Chemistry La Sapienza University of Rome Roma Italy; ^3^ Istituto di Struttura della Materia‐CNR (ISM‐CNR) Via del Fosso del Cavaliere Roma Italy; ^4^ Université Claude Bernard Lyon 1, CNRS, IRCELYON, UMR 5256 Villeurbanne Cedex France; ^5^ Iritaly Trading Company S.r.l. Roma Italy; ^6^ Department Electronics Engineering University of Rome “Tor Vergata” Roma Italy

**Keywords:** carbon nanotubes, electrochemical reactions, methanol production, porphyrinoids, sustainable synthesis

## Abstract

We report a sustainable and cost‐effective synthesis of cobalt phthalocyanine (CoPc) using low‐impact solvents and a streamlined purification procedure, achieving an 85% yield with limited waste generation, as indicated by an environmental factor (E‐factor) of 18.6. This route provides an eco‐friendly approach to producing a molecular electrocatalyst for ${\mathrm{CO}}_{2}$ reduction. The catalytic activity of the synthesized CoPc is validated by its integration into a gas diffusion electrode with carbon nanotube supports, exhibiting methanol production with a Faradaic efficiency up to 12%, matching the values reached with the conventionally synthesized counterparts. Notably, under high loading (micrometric crystallites of CoPc), ${\mathrm{CO}}_{2}$‐to‐CO conversion reached 65%, in fair alignment with literature benchmarks. Our work shows that green synthesis can yield high‐performance catalysts for ${\mathrm{CO}}_{2}$ electrochemical reduction (${\mathrm{CO}}_{2}\mathrm{RR}$), enabling sustainable methanol production.

## Graphical abstract

A sustainable synthesis of cobalt phthalocyanine (CoPc) using low‐impact solvents yields high‐performance electrocatalysts with reduced waste (E‐factor 18.6). When integrated into gas diffusion electrodes, CoPc achieves up to 12% Faradays efficiency for CO_2_‐to‐methanol and 65% CO_2_‐to‐CO conversion, aligning with state‐of‐the‐art materials and enabling greener CO_2_ reduction (CO_2_RR).

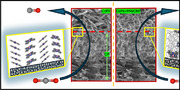

## 1 Introduction

Carbon dioxide (${\mathrm{CO}}_{2}$) as the most abundant greenhouse gas is a major contributor to global warming [1]. In this context, ${\mathrm{CO}}_{2}$ capture and utilization technologies [2] are an attractive approach to mitigate ${\mathrm{CO}}_{2}$ emissions and valorize it into useful molecular energy carriers such as methane [3], urea [4, 5, 6, 7, 8], formic acid [9], and alcohols [10, 11, 12, 13, 14]. In particular, among alcohols, methanol (MeOH) stands out as a beneficial liquid and environmentally‐safe fuel option, as well as strategic intermediate of low‐carbon chemical value‐chains [15], that can reduce the dependence on fossil fuels and promote the use of renewable energy.

There are several catalytic methods to synthesize methanol from ${\mathrm{CO}}_{2}$, such as plasma‐assisted conversion [16], hydrogenation [17, 18], photoelectrochemical reduction [19], and electrochemical reduction (${\mathrm{CO}}_{2}\mathrm{RR}$) [20, 21, 22, 23, 24, 25, 26, 27, 28, 29]. ${\mathrm{CO}}_{2}\mathrm{RR}$ is a promising process that converts carbon dioxide into value‐added chemicals at the surface of an electrocatalyst. The process involves the use of water as the hydrogen source instead of $H_{2}$ and it is performed under ambient pressure and temperature. These features make it a sustainable and attractive approach for ${\mathrm{CO}}_{2}\mathrm{valorization}$ and conversion, for instance, into methanol. However, achieving high efficiency in this conversion remains a significant challenge as it requires the transfer of six electrons and six protons per molecule of ${\mathrm{CO}}_{2}$. More specifically, the conversion typically proceeds via a carbon monoxide intermediate (CO*) formed through the reduction of adsorbed ${\mathrm{CO}}_{2}$ on the catalyst surface [30]. Thus, when methanol is the desired product, a key challenge lies in promoting the further reduction of the CO* intermediate rather than its desorption or coupling with another CO* to form multi‐carbon products such as ethylene [31]. Achieving this level of control is particularly difficult, as it demands not only effective stabilization of the CO* intermediate but also precise tuning of the catalyst to suppress competing pathways. As a result, the electrochemical reduction of ${\mathrm{CO}}_{2}$ to methanol is often characterized by sluggish kinetics and limited selectivity toward the desired product. These challenges make the identification and development of catalysts capable of favoring the full ${\mathrm{CO}}_{2}$‐to‐methanol pathway especially demanding, and underscore the need for molecular‐level control over catalyst structure and environment.

Several first‐row transition metal complexes, such as those based on cobalt (Co), iron (Fe), and nickel (Ni), have been shown to facilitate the production of methanol in organic solvents when used as heterogeneous or homogeneous electrocatalysts [32]. In particular, the M‐$N_{4}$ square‐planar active site found in metal phthalocyanines or single‐atom catalysts dispersed on nitrogen‐doped carbon has yielded some of the most significant results in ${\mathrm{CO}}_{2}$ electroconversion to methanol in recent years. The use of cobalt and copper, both abundant and economically favorable transition metals [33], in Co‐$N_{4}$ and Cu‐$N_{4}$ active sites successfully stirred the selectivity of ${\mathrm{CO}}_{2}$ RR toward methanol.

A series of recent studies on CoPc have shown the importance of using multi‐walled carbon nanotubes (MWCNTs) as support to disperse the molecular catalyst and achieve high methanol selectivity. [34, 35, 36]. The key role of this support is however still a subject of debate as selectivity toward methanol is highly sensitive to the electrode preparation process and electrochemical operating conditions. Factors such as electrolyte composition [37], electrolyte flow rate [38], phthalocyanine stacking [39], and functionalization [35] can influence electrocatalytic selectivity toward the production of methanol. In particular, aggregate formation and strain are two factors that affect the performance of CoPc on CNTs. CoPc dissolves and conforms more readily over SWCNT or MWCNTs (single or multiwall carbon nanotubes) with different diameters. In fact, biaxial strain can improve methanol selectivity [40]. Recently, it has been reported that ball milling of CoPc and carbon nanotubes can enhance methanol formation [41].

While the systems described above have achieved promising selectivity toward methanol production, most research to date has concentrated on improving catalytic efficiency and activity. In contrast, considerably less attention has been given to the sustainability of catalyst synthesis itself, including the environmental impact of reagents, solvents, and processing steps used in fabrication. Yet, ensuring that the materials employed in ${\mathrm{CO}}_{2}$ reduction technologies are sustainable, both environmentally and economically, is essential for their long‐term viability. The full lifecycle of catalyst materials, encompassing synthesis, processing, and end‐of‐life management, can significantly influence the net environmental benefit of ${\mathrm{CO}}_{2}$ conversion. Without accounting for these aspects, the net sustainability of ${\mathrm{CO}}_{2}$ conversion technologies may be reduced, particularly when considering the full material and energy footprint of catalyst deployment. Here, we aim to address this gap by placing sustainable catalyst synthesis at the core of ${\mathrm{CO}}_{2}\mathrm{RR}$ research by developing a greener synthetic route for cobalt phthalocyanine, designed to minimize environmental impact while maintaining compatibility with electrocatalytic applications. The synthesis employed environmentally benign solvents such as glycerol and anisole, coupled with low‐impact purification techniques. This methodology significantly reduced reaction waste and production costs, thereby demonstrating the feasibility of sustainable material synthesis in advancing ${\mathrm{CO}}_{2}$ reduction technologies, an aspect that remains largely under explored in the field of ${\mathrm{CO}}_{2}\mathrm{RR}$. To validate the electrocatalytic activity of the green‐synthesized CoPc, we dispersed it onto MWCNTs, incorporated the resulting CoPc/MWCNT hybrid into a gas diffusion electrode, and evaluated its performance for ${\mathrm{CO}}_{2}$ reduction to methanol. In addition, a CoPc electrode without nanotubes was prepared to assess the behavior of the green‐synthesized catalyst in a minimal‐support configuration, providing meaningful insights into partial ${\mathrm{CO}}_{2}$ reduction processes. In particular, the selective formation of CO under these conditions highlights the potential utility of CoPc in two‐step ${\mathrm{CO}}_{2}$ valorization strategies [42], where CO is first produced electrochemically and subsequently converted to methanol or other multi‐electron products in a downstream reactor.

A comprehensive analysis of the structural, chemical, and electrochemical changes induced by the interaction between CoPc and carbon‐based substrates confirms that the catalyst retains its essential properties, supporting its integration into ${\mathrm{CO}}_{2}$ conversion technologies with reduced environmental impact.

## 2 Experiment and Methods

### 2.1 General Procedure for CoPc Synthesis

All reagents and solvents were purchased from Merck Life Science S.r.l. and Carlo Erba Reagents, and were used without further purification. Glycerol was stored over 3 Å molecular sieves. 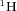 NMR spectra were recorded on a Bruker AVANCE 600 NMR spectrometer operating at a proton frequency of 600.13 MHz; chemical shifts are given in ppm relative to tetramethylsilane (TMS), employed as internal standard. FTIR spectra were recorded using a Shimadzu FT‐IR Prestige‐21 spectrometer equipped with an attenuated total reflectance (ATR) unit. UV‐Vis spectra were acquired in transmission mode using a double‐beam Perkin‐Elmer Lambda 950 UV‐Vis/NIR spectrophotometer. Measurements were conducted in THF with 1 cm pathlength quartz cuvettes, using a blank solvent cuvette in parallel as reference. High‐resolution mass spectroscopy (HRMS) measurements were performed using an Orbitrap Exploris 120 mass spectrometer (ThermoFisher Scientific) coupled to an atmospheric pressure matrix‐assisted laser desorption/ionization (AP‐MALDI (ng)) ultra‐high resolution (UHR) ion source (MassTech Inc.). Instrumental setup and sample preparation are described elsewhere [43]. Briefly, approximately 1 mg of the synthesized compound was added to 200 μL of acetonitrile and sonicated for a few minutes in an ultrasonic bath, yielding a colored suspension. A 1 μL aliquot of the resulting suspension was then spotted onto the wells of the MALDI target plate. The sample was then covered with an equal volume of a 5 mg/mL solution of alpha‐cyano‐4‐hydroxycinnamic acid (CHCA) prepared in acetonitrile/ultrapure water/trifluoroacetic acid 50/50/0.1% and used as a MALDI matrix for positive‐ion mode acquisition. After solvent evaporation, sample was irradiated with a UV laser (ν= 355 nm) operating in spiral motion mode at a repetition rate of 1000 Hz and 1% laser energy. Other instrumental parameters, such as Ionization Voltage (V) and Ion Transfer Tube Temperature (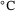), were optimized to maximize the ionic intensity of the compound of interest. The mass spectrum was acquired over the m/z range of 230‐1000 Da with a mass resolution of 1,20,000 at m/z 200 and an injection time of 100 ms. The instrument was calibrated daily using the Flexmix Calibration solution (ThermoFisher Scientific), while fluorantrene served as an internal lock mass via activation of the EASY‐IC source option allowing for scan‐to‐scan mass scale recalibration during each analysis. Raw data were processed using FreeStyle software v. 1.8 (ThermoFisher Scientific) included in the Xcalibur package. Cyclic voltammetry (CV) was performed in anhydrous DMF using a single‐compartment, 15 mL electrochemical cell. Prior to the measurements, the electrolyte solution was purged with nitrogen for at least 15 min to ensure an oxygen‐free environment. Continuous $N_{2}$ flow was maintained during the measurements to prevent reoxygenation. A glassy carbon disk electrode was used as the working electrode. The reference electrode was a non‐aqueous Ag/AgCl/DMF electrode, and a platinum coil served as the counter electrode. The reported potential is referenced to the Ag/AgCl/DMF electrode. Cyclic voltammograms were recorded at a scan rate of 20 mV/s.

In a two‐necked 25 mL round‐bottom flask equipped with a reflux condenser and magnetic stir bar, 1.0 g (1.0 eq) of phthalonitrile and the chosen amount of 1,8‐diazabicyclo[5.4.0]undec‐7‐ene (DBU) were dissolved in a 10:1 (w/w) mixture of glycerol and anisole and stirred vigorously. Separately, 0.26 eq of cobalt(II) acetate tetrahydrate was pre‐treated on a hot plate for 5 min until the color changed from pink to violet. It was then added to the reaction mixture in one portion. The mixture was then heated at 165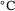 for 6 h, resulting in a dark blue suspension. After cooling to room temperature, the mixture was quenched by the addition of 1.0 mL of 1 M HCl followed by 10.0 mL of distilled water. The suspension was sonicated in an ultrasound bath for a few minutes and then filtered under vacuum. The resulting solid was thoroughly washed with distilled water (10.0 mL) and dried under vacuum. To assist in the removal of polar and water‐soluble impurities remaining after acid treatment, the dried powder was suspended in 10.0 mL (7.9 g) of methanol and stirred at 50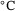 for 15 min. This step acts as a pre‐purification prior to Soxhlet extraction. The methanol used in this step was collected, stored, and later combined with the Soxhlet extraction solvent for recovery via distillation. After the prewash, the solid was again dried under vacuum. Final purification was carried out using Soxhlet extraction with 150.0 mL (118.8 g) of methanol, continued for 8 h until the extract became nearly colorless . The methanol from both the Soxhlet extraction and the prewash was recovered by distillation, with an average recovery yield of 90% (corresponding to 114.0 g), and reused in subsequent preparations. Under the best‐performing conditions, summarized in Table 1, 941 mg (85% yield) of CoPc were obtained. IR (neat, ${\mathrm{cm}}^{-1}$): 1609–1589 (w, C = C stretching), 1523 (m, C = N stretching), 1332 (m, C–N stretching), 729 (vs, C‐H out of plane)). UV/Vis (THF, nm): λmax = 655 (Q band), 594 (shoulder), 323 (Soret band). HRMS (AP‐MALDI) m/z calcd for $\left[[C_{32}H_{16}N_{8}\mathrm{Co}]\right]$ 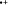: 571.08354 ${\left[M\right]}^{•+}$; found: 571.0837.

**TABLE 1:** Reaction parameter variations for the synthesis of CoPc. All reactions were carried out using 0.26 equivalents of cobalt acetate tetrahydrate, which was thermally pre‐treated on a hot plate prior to its addition to the reaction mixture, and a reaction time of 6 h. The best‐performing conditions are highlighted in bold.

**Solvent amount (g)**	**DBU (eq)**	**Yield**
6.9	0.013	78
5.5	0.013	74
4.4	0.013	75
**3.3**	**0.013**	**85**
3.3	0.013	59^*^
3.3	0.051	71
3.3	0.13	67
3.3	0.00	70

^*^ Reaction performed in air atmosphere.

### 2.2 Ink Preparation

MWCNTs (>98% purity, supplied by Merck, reference 698849‐1G) were first heated at 500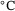 for 1h in air, stirred in 5 M HCl overnight, centrifuged and rinsed with deionized water, and finally dried under vacuum. CoPc was supported on them according to the following procedure. First, 30 mg of MWCNT were suspended and stirred inside 30 mL CoPc solution in HPLC‐grade (>99.9% purity, Merck) DMF at 0.05 mg/mL for two days. The suspension was then centrifuged, the CoPc+MWCNT powder collected, rinsed with DMF, and suspended again. This was repeated until the supernatant was colorless for two consecutive rinses. Then a final rinse with ethanol was performed before drying the powder under vacuum at 60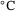. Inductively coupled plasma (ICP) spectroscopy revealed a cobalt loading of 0.5 ± 0.1 % in cobalt was achieved through this procedure (based on four repeat experiments). The loading was not further optimized for this study.

### 2.3 Electrode Preparation

First, an ink was prepared by mixing 5 mg of CoPc electrocatalyst powder (either pure CoPc or CoPc+MWCNT composite) and 5 μL of Nafion® solution (5 v% in water/isopropanol, supplied by Merck) in 1 mL of ethanol. The ink was sonicated for 30 min in an ice bath and then sprayed onto a square porous carbon paper (CP, AvCarb GDS5130) of 5 ${\mathrm{cm}}^{2}$. The spray was performed with a commercial airbrush system and the substrate was heated at 60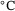 during the process. The electrode was then dried overnight at 80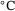. The final electrocatalyst loading was in the 0.9 – 1.2 mg/${\mathrm{cm}}^{2}$ range, as determined by weighing the final electrode on a precision scale. For electrochemical experiments, each electrode was cut into smaller pieces of roughly 1×2.2 ${\mathrm{cm}}^{2}$.

### 2.4 Electrochemical Testing

Electrochemical measurements were performed inside a commercial airtight jacketed H‐type electrochemical cell (from DekResearch). Both compartments of the cell were separated by a proton exchange membrane (Nafion® N‐117). Both compartments were filled with 0.5 M ${\mathrm{KHCO}}_{3}$ (pH 8.4) and all electrochemical experiments were performed with a conventional three‐electrode setup connected to a potentiostat (OrigaLys OGF01A). The working electrode containing CoPc electrocatalysts and the Ag/AgCl reference electrode (3.5 M KCl) were located in the cathode chamber, whereas the Pt coil counter electrode was placed into the anode chamber. The surface area of electrocatalytic layer of the working electrode exposed to the electrolyte was adjusted to 1 ${\mathrm{cm}}^{2}$. The catholyte was continuously sparged with a 50 mL/min ${\mathrm{CO}}_{2}$ flow. The ohmic drop (typically between 4 and 5 Ω) was measured before each experiment and used to correct the electrochemical potential of the working electrode in cyclic voltammetry and chronoamperometry experiments.

### 2.5 Product Analysis

Gas products were analyzed in real time by using an on‐line microgas chromatograph (INFICON Micro GC Fusion) equipped with a molecular sieve column, a polymer PLOT‐Q column and thermal conductivity detectors (TCD). NMR experiments were performed to analyze liquid products by collecting 0.1 mL of electrolyte, which was mixed with 0.4 mL of $D_{2}O$ in an NMR tube. Samples were then analyzed on a Bruker 400 MHz NMR spectrometer, and data were processed in TopSpin 3.6.5. A water peak suppression procedure was applied to each sample. For the quantification of methanol, an internal standard of DMSO of known concentration was added to the NMR tube. A typical spectrum is presented in Figure S14.

### 2.6 Samples Characterization by SEM/EDX, XRD, and XPS

Scanning Electron Microscopy (SEM) experiments were conducted using a TESCAN Analytics VEGA instrument. Elemental maps from energy‐dispersive X–ray spectroscopy (EDX) were obtained using an Xplore detector from Oxford Instruments. X‐ray diffraction (XRD) measurements were carried out using a RIGAKU SmartLab SE in Bragg–Brentano mode and low–angle configuration (2θ = 2
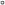
–20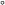) with a fixed knife edge. A high–resolution, ultrafast 1D X–ray detector (D/teX Ultra250, 0/1D) was used for data acquisition. Peak fitting was performed using SmartLab Studio II software. X–ray photoelectron spectroscopy (XPS) studies were performed using an ultra–high vacuum (UHV) instrument equipped with aluminum Kα X–ray source (hν = 1486.6 eV) and a five–channeltron (VG 150 mm mean radius) hemispherical electron analyzer. Experimental spectra were deconvoluted by using Voigt functions with a Shirley background, as fitting functions.

## 3 Results

### 3.1 Cobalt Phthalocyanine Synthesis and Ink Preparation

**FIGURE 1:**
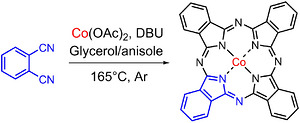
Synthesis of CoPc using cobalt acetate as templating salt and 1,8‐diazabicyclo[5.4.0]undec‐7‐ene (DBU) as the base, with a reaction medium composed of glycerol and anisole at a 10:1 w/w ratio.

To align more closely with sustainable research practices, we pursued an in‐house synthesis of cobalt phthalocyanine by developing and implementing a low‐impact protocol. This approach is part of a broader research effort by our group to develop sustainable synthetic protocols based on benign solvents such as glycerol and anisole, as recently demonstrated in various context of synthetic chemistry [44, 45, 46]. Although cobalt phthalocyanine is readily available from commercial sources, we opted to establish our own synthetic route to ensure greater control over the process and its environmental footprint. This decision was driven by multiple considerations. The adopted procedure employs renewable solvents and streamlined purification steps, substantially reducing chemical waste and avoiding toxic reagents. This approach aligns with several key green chemistry principles, including the use of safer solvents, waste prevention, and design for energy and material efficiency. Additionally, conducting the synthesis at the laboratory scale provides tighter control over product purity, helping to eliminate variability and contamination, critical factors when preparing catalysts for electrocatalytic applications. Among the available precursors, phthalonitrile was selected due to its ability to afford phthalocyanines with high atom economy, a key green chemistry metric that reflects the proportion of reactant atoms incorporated into the final product. In the case of phthalonitrile, all atoms from the organic precursor are retained within the macrocyclic phthalocyanine structure, as highlighted in Figure 1. The cyclotetramerization of four phthalonitrile units therefore proceeds without the elimination of small organic byproducts, generating minimal molecular waste. As such, the overall atom economy of the process is dictated by the nature of the metal salt only, and the phthalocyanine formation occurs with high efficiency and minimal stoichiometric byproducts, making this synthetic route particularly appealing from a green chemistry standpoint. Cobalt phthalocyanine was synthesized using a glycerol‐anisole solvent mixture, offering a more environmentally sustainable alternative to solvents such as N,N‐dimethylaminoethanol (DMAE), nitrobenzene, and quinoline, typically employed for this purpose. Indeed, both glycerol and anisole are derivable from renewable sources, non‐toxic and favorably ranked in solvent selection guides. Briefly, phthalonitrile was dissolved in the solvent mixture in the presence of cobalt acetate and DBU and subsequently allowed to react at 165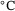 for 6 h. The reaction scheme is illustrated in Figure 1. Taking advantage of the limited solubility of unsubstituted phthalocyanine in common organic solvents, purification was efficiently achieved through sequential washing with water followed by filtration. A preliminary hot methanol wash was introduced prior to Soxhlet extraction to selectively remove highly soluble impurities and reduce the impurity load entering the extractor. This step improves the efficiency and sustainability of the Soxhlet purification by minimizing the number of required cycles and solvent usage. The experimental protocol was initially optimized based on the conditions reported in [44] focusing on solvent volume and maintaining a reaction temperature of 165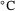. While increasing the solvent amount led to only a modest improvement in yield, a more significant enhancement was observed when reducing the amount to 3.3 g of solvent mixture per 1.0 g of phthalonitrile; further reduction led to poor stirring and an inhomogeneous reaction environment, potentially compromising reproducibility under the selected conditions. An inert atmosphere was found to be essential for achieving high yields, as only 59% yield was obtained under air in the solvent‐optimized conditions. This is consistent with the known reactivity of oxygen, which can induce side reactions by oxidizing the nitrile groups of phthalonitrile, thereby decreasing the amount of precursor available for cyclotetramerization. The influence of base concentration was subsequently evaluated. Increasing the DBU content from 1 mol% to 10 mol% did not lead to any improvement in yield; on the contrary, it resulted in a decrease from 85% to 67%. When no DBU was employed, a 70% yield was achieved confirming that a small amount of base is beneficial for the reaction outcome. The decreased yield at higher DBU concentrations is likely due to the promotion of side reactions such as partial hydrolysis of the nitrile groups, oligomerization, or degradation of glycerol under strong basic conditions at the chosen temperature. These effects can interfere with the cyclotetramerization process, highlighting the importance of maintaining low base levels to ensure high selectivity and efficiency. Notably, the ability of the reaction to proceed efficiently with only catalytic amounts of DBU is a clear advantage in terms of waste reduction and alignment with green chemistry principles. All the tested conditions are summarized in Table 1.

The formation of cobalt phthalocyanine was confirmed by a combination of 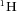 NMR, MALDI mass spectrometry, Infra‐red (IR) and UV‐Vis spectroscopy, providing evidence of successful metal coordination and structural integrity of the macrocycle. The purity of the synthesized CoPc was verified by recording its 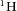 NMR spectrum in DMSO‐*d* 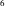 and comparing it with those of phthalonitrile and phthalimide, the two main possible contaminants. Although the scarce solubility of CoPc in organic solvents and the paramagnetic nature of Co^2+^ prevent the use of 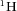 NMR for accurate peak integration or detailed structural elucidation, the spectrum remains useful for assessing sample purity. As expected, the CoPc spectrum showed broad solvent‐related signals and a weak broad singlet at 10.29 ppm that may be tentatively assigned to macrocyclic aromatic protons, although it is noticeably deshielded compared to those reported for other unsubstituted metallophthalocyanines [44]. Conversely, no resonances corresponding to phthalonitrile, phthalimide, or oligomeric aromatic byproducts were detected. In contrast, when a mixture of CoPc intentionally contaminated with micromolar amounts of phthalonitrile and phthalimide was analyzed, the characteristic signals of the contaminants were clearly visible. The corresponding spectra and experimental details are provided in the Supplementary Information (SI). The IR spectrum, reported in Figure S10 shows no signals above 1610 ${\mathrm{cm}}^{-1}$, consistent with the formation of an unsubstituted phthalocyanine. Notably, the absence of bands in the 3400–3100 ${\mathrm{cm}}^{-1}$ region indicates the lack of N–H stretching vibrations, confirming that no free‐base phthalocyanine is present in the sample. Additionally, the strong absorption band observed at 729 ${\mathrm{cm}}^{-1}$, attributed to C‐H out‐of‐plane bending and Pc ring deformations, further supports the successful phthalonitrile cyclotetramerization. The absence of signals in the 2200–2100 ${\mathrm{cm}}^{-1}$ and 1700 ${\mathrm{cm}}^{-1}$ regions confirms that nitrile and carbonyl functional groups are no longer present. Together, these findings indicate an efficient conversion of the phthalonitrile precursors into the metalated phthalocyanine and effective removal of potential byproducts, such as phthalimides, during purification. Complementary information is provided by the UV‐Vis transmission spectrum of CoPc in tetrahydrofuran (THF), reported in Figure S9, which shows the characteristic Q‐band centered at 655 nm, accompanied by a shoulder at 594 nm, and a less intense Soret band at 323 nm. This spectral profile is typical of cobalt phthalocyanine and confirms the expected electronic structure of the macrocycle in solution. The AP‐MALDI mass spectrum further supports this assignment, showing the characteristic peak corresponding to the CoPc radical cation at m/z 571.0825.

The environmental sustainability of chemical syntheses can be assessed using the Environmental‐factor (E‐factor), a green chemistry metric defined as the ratio of waste mass to product mass [47] (see Supporting Information). Low E‐factor values indicate minimal waste generation and are considered desirable. While it is just one of many factors to consider when evaluating the overall environmental sustainability of a synthetic process, the E‐factor provides valuable insights into waste management through a simple ex‐post analysis of readily available data. For the synthesis of CoPc, we obtained an E‐factor of 18.6, which is among the lowest reported for lab‐scale syntheses of phthalocyanines and highlights the efficiency of the procedure in terms of waste minimization. [48, 49]. Additionally, the estimated synthesis cost of CoPc, determined following the methodology described by Osedach et al. [50], was as low as 0.48 €/g (Table S1 and S2). While we acknowledge that such estimations do not consider personnel, facility, energy, and maintenance costs, our synthetic approach enables a substantial reduction in CoPc cost compared to commercial suppliers, whose average prices range from €7.24 to €20.91 per gram (Table S2).

To evaluate the electrocatalytic potential of the green‐synthesized cobalt phthalocyanine, we prepared two electrode formulations: one in which CoPc was dispersed over MWCNTs, and another in which CoPc was directly deposited onto a carbon paper (CP) substrate. Both systems were fabricated via spray‐coating using catalyst inks of tailored composition. Specifically, one ink consisted of CoPc, Nafion®, and isopropyl alcohol (referred to as CoPc), while the other included additional MWCNTs (referred to as CoPc+MWCNT). For comparison, control electrodes based on CP and MWCNT/CP—processed under identical conditions but without CoPc—were also characterized. The physical and structural features of the resulting catalytic systems were investigated using SEM, XRD, and XPS analyses, as discussed in the following sections.

### 3.2 CoPc+MWCNT/CP Sample

**FIGURE 2:**
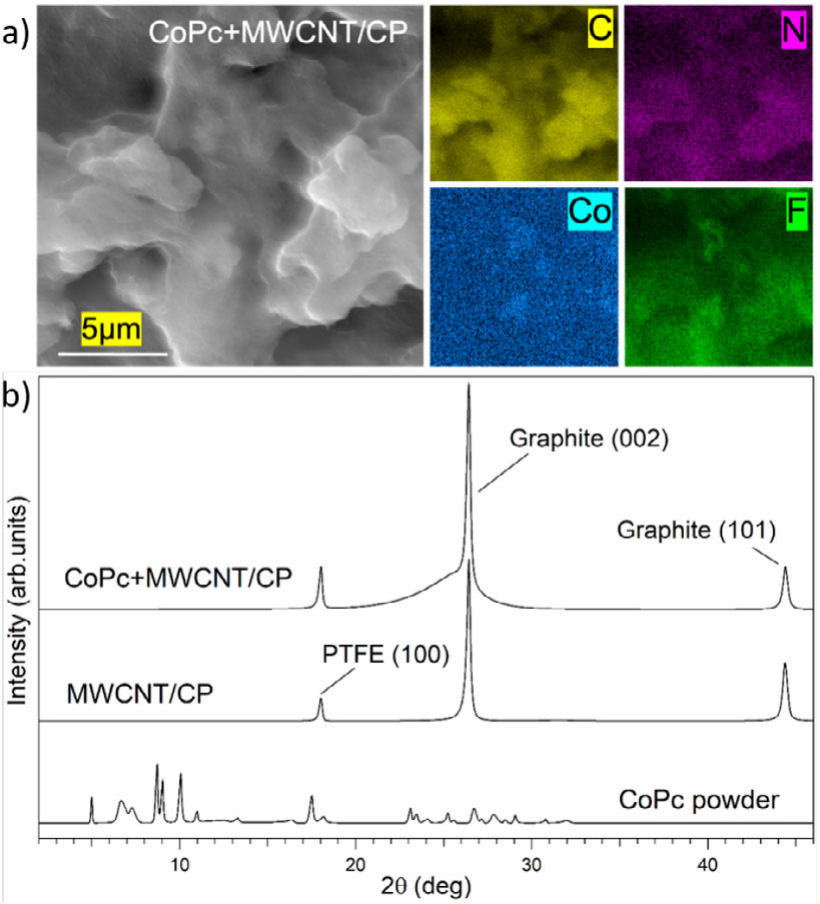
Panel (a): SEM image (20kX magnification) of the CoPc+MWCNTs/CP (left) along with the EDX maps for C, N, Co, and F elements (right). Panel (b), XRD pattern for a powder of CoPc molecules, MWCNT/CP and CoPc+MWCNT/CP samples.

In Figure 2 the SEM image taken on the sample treated with CoPc+MWCNTs ink is displayed, along with the corresponding EDX maps of C, N, Co, and F elements. Although the spatial resolution of the EDX maps does not allow the nanotubes to be singled out, the carbon signal aligns with the cloud‐like features observed in the SEM image. Nitrogen and cobalt maps directly relate to the presence of CoPc molecules, and display the same features as the SEM images and the carbon map. Fluorine was also detected, and attributed to the Nafion® binder and the PTFE treatment of the commercial CP surface.

Figure 2b shows the XRD patterns of the bare and functionalized substrates, along with reference data collected from CoPc powder. The MWCNT/CP sample shows no additional crystalline features beyond those observed for the CP substrate (see Figure 4), indicating that the nanotubes do not significantly alter the bulk crystalline signature of the underlying support. Similarly, the XRD pattern of the CoPc+MWCNT/CP sample does not show any additional peaks attributable to crystalline CoPc, suggesting that under these conditions, CoPc is well dispersed over the nanotube network.

**FIGURE 3:**
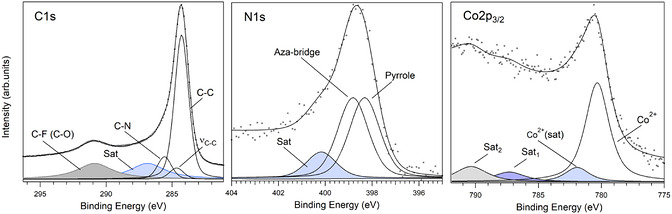
C1s, N1s, and Co2p core level spectra recorded on the CoPc+MWCNTs/CP sample.

**FIGURE 4:**
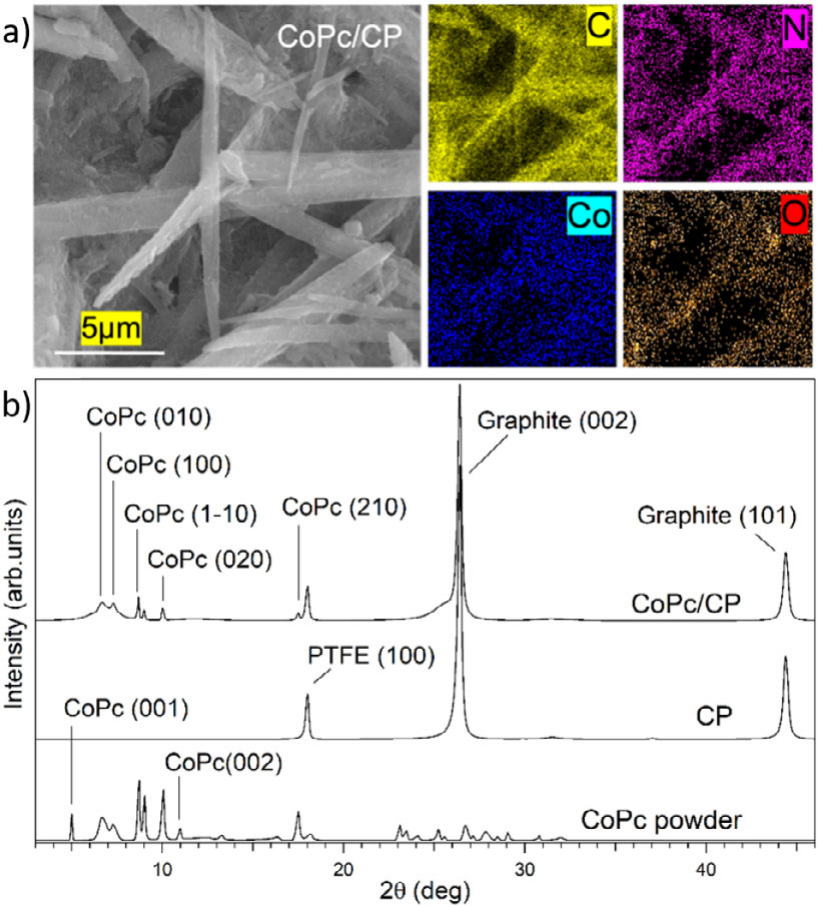
Panel (a): SEM image (20kX magnification) of the CoPc/CP (left) along with the EDX maps for C, N, Co, and O elements (right). Panel (b), XRD pattern for a powder of CoPc molecules, CP and CoPc/CP samples.

The XPS wide‐scan spectra of the two CoPc‐coated samples, along with the reference spectra from bare CP and MWCNTs/CP, are displayed in Figure S1. Fluorine was detected, confirming the incorporation of PTFE in agreement with the results obtained via EDX analysis. The measured XPS spectra indicate that the CoPc molecules retain their chemical integrity upon spray‐coating of the ink, with no signs of degradation.

A peak fitting to the most relevant core level data was performed (Figure 3). In the C1s region, the main peak is attributed to aromatic C–C bonds within the benzene rings. Additional components were observed at higher binding energies (BE), such as those corresponding to the C‐N bond and vibrational coupling ($\nu_{C-C}$) [51], see Table 2. Two additional components are needed and assigned to the C‐C and C‐N shake‐up peaks named ‘ *Sat* ’ in the C1s panel, in agreement with the literature [51, 52, 53]. It is also worth considering that components associated with the presence of PTFE may be present [54], as supported by the detection of fluorine and oxygen in the wide‐scan XPS spectra (Figures S1 b) and c)) this is reason why new components appear in the C1s core levels above 290 eV BE, likely corresponding to C‐O [55] and C‐F bonds [54]. The appearance of components at higher BE may alternatively be attributed to surface oxidation phenomena, such as the formation of C–O or C = O bonds due to interactions with ambient air [56].

**TABLE 2:** Binding energies (eV) of the different components of the C1s, N1s, and Co2p core level spectra taken on CoPc/CP and CoPc+MWCNT/CP samples.

C1s	C–C	C–N	$\nu_{C-C}$	Shake‐up	
				C–C	C–N
CoPc/CP	284.6	285.9	284.9	287.5	292.3
CoPc+MWCNT/CP	284.3	285.6	284.7	286.9	290.9

N1s	Pyrrole	Aza–bridge	Sat		
CoPc/CP	398.6	399.1	400.5		
CoPc+MWCNT/CP	398.3	398.8	400.2		

Co2p	${\mathrm{Co}}^{2+}$	${\mathrm{Co}}^{2+}$(Sat)	${\mathrm{Sat}}_{1}$	${\mathrm{Sat}}_{2}$	
CoPc/CP	780.5	782.2	787.5		
CoPc+MWCNT/CP	780.4	781.9	787.3	790.4	

The N1s spectrum in Figure 3 was deconvoluted by using two main components, corresponding to the pyrrole and aza‐bridge groups, along with an additional component at higher BE. We employed two components with an equal full width at half maximum (FWHM) of 1.3 eV, consistent with the typical resolution of the instrument. The energy shift between the main components is approximately 0.5 eV, in agreement with the literature [52, 57, 58, 59]. The component labeled ‘ *Sat* ’ is attributed to shake‐up satellites [52, 58, 60].

Regarding the Co2p_3/2_ spectrum, the most intense component is detected at about 781 eV BE, which could, in principle, be attributed to Co^3+^ oxidation state [56]. As a consequence, such a component would be then associated to the formation of an out‐of‐plane Co–O bond (with respect to the macrocycle), potentially influenced by oxygen‐containing functional groups present on the carbon nanotube surface. Indeed, the O1s signal around 530 eV suggests the presence of minor oxidation species, such as hydroxyl and carbonyl groups. However, their concentration appears insufficient to induce a significant conversion of Co^2+^ to Co^3+^. Moreover, while interactions with the nanotubes may slightly modify the chemical environment of the cobalt center, they do not appear to significantly alter its oxidation state. As a matter of fact, the Co^2+^ oxidation state in methanol formation has been shown to be catalytically relevant, as demonstrated in Ref. 61, where molecular distortion of CoPc facilitates charge accumulation at the metal center and suppresses CO desorption. Based on these considerations, we are inclined to attribute the additional spectral feature to a satellite state associated with the Co^2+^ component, rather than to a distinct Co^3+^ species [62].

All XPS spectra exhibit a slight shift toward lower BE; however, no *relative* shift is observed among the individual core levels. Interestingly, similar phenomena have been reported in the literature regarding the dispersion of CoPc molecules on carbon nanostructures. For instance, Huai et al. [53] observed a slight shift toward lower BE in the N1s and Co2p spectra upon dispersion on MWCNTs, whereas Su et al. [36] reported shifts in the opposite direction. These contrasting results highlight the sensitivity of core‐level BE to subtle variations in the local chemical environment and the dispersion state of the catalyst. The BE positions of the various components are listed in Table 2.

### 3.3 CoPc/CP Sample

In Figure 4 SEM image shows the effect of the CoPc‐based ink without carbon nanotubes deposited on the bare carbon–based gas diffusion layer. The deposition of CoPc on the CP substrate leads to the formation of a distinct needle‐shaped texture (CoPc/CP panel) made of micro‐metric crystallized structures that are indicative of strong CoPc–CoPc interactions and limited dispersion on the CP surface. Besides the SEM image, the corresponding EDX maps for C, N, Co and O elements are displayed. While the carbon signal can be ascribed to both the CP substrate and CoPc molecules, Co and N maps clearly follow the needle‐like morphology, confirming their relation with the CoPc crystallites. Areas with a higher oxygen content can be ascribed to surface contamination rather than inherent features of the CoPc structure.

The CP substrate exhibits three characteristic peaks at 18.2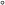, 26.6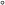 and 44.5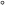, corresponding to the (100) plane of PTFE and the (002) and (101) planes of graphite, respectively. The CoPc powder displays distinct reflections indexed to the (210), (002), (020), ($1\bar{1}0$), (100), (010), and (001) planes, in agreement with crystallographic data reported in the literature [63, 64], confirming its high degree of crystallinity. The CoPc/CP sample presents a diffraction pattern that combines features of both the CoPc powder and the CP substrate. This overlap confirms the crystalline nature of the CoPc coating and correlates well with the formation of the micrometric, needle‐like CoPc aggregates observed in SEM imaging (Figure 4).

**FIGURE 5:**
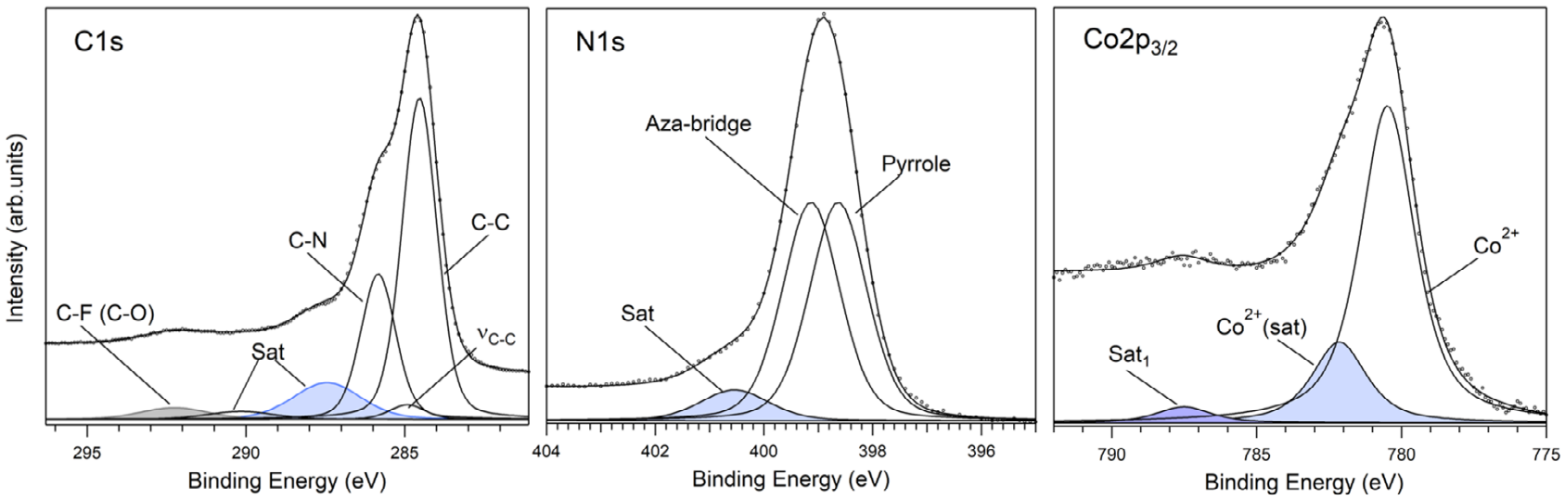
C1s, N1s, and Co2p core level spectra recorded on the CoPc/CP sample.

The core levels reported in Figure 5 complement the wide scans reported in Supporting Information, Figure S1. It is noticed that due to the aggregate nature of the CoPc, the intensity of the peaks is larger than the MWCNTs‐dispersed case, as expected. The spectra were deconvoluted by using the same peak fitting procedure performed on the data recorded on the samples containing MWCNTs, relying on the ability of the CoPc molecule to maintain its chemical integrity. The devonvolution reported by Papageorgiou et al. [51] for the C1s peak was used and the results listed in see Table 2. With respect to the CoPc+MWCNTs/CP sample, the component assigned to the C–N bond appears with relatively higher intensity. Even for the N1s, the deconvolution shows no exception: the data are well described by the three components already listed above, that is, pyrrole aza‐bridge and shake up satellite.

The most intense peak of the Co2p_3/2_ core level is attributed to the Co^2+^ owing to the same reasons discussed in the case of the MWCNTs. Indeed, the hypothesis of an out‐of‐plane oxygen atom bonded to the Co center of the CoPc molecules located on the surface of the crystallites, becomes even less likely: due to a more favorable surface‐to‐bulk ratio in the case of dispersed CoPc (as there is no bulk CoPc in the presence of MWCNTs), the hypothetical Co^3+^/Co^2+^branching ratio would be expected to be much higher. This is why we ascribe the two most intense components to the Co^2+^ oxidation state and its satellite. It is also worth noting that in the Co2p core level of Figure 5 no satellite “${\mathrm{Sat}}_{2}$” is present, suggesting an attribution related to the carbon nanotubes only.

The core levels exhibit a consistent rigid shift toward lower BE, larger than the case of the carbon nanotubes. We tentatively attribute this shift to a reduced electron refilling efficiency following photoelectron ejection, possibly due to the less metallic nature of the CoPc crystallites, which may lead to a localized charging effect.

### 3.4 Electrochemical Testing

**FIGURE 6:**
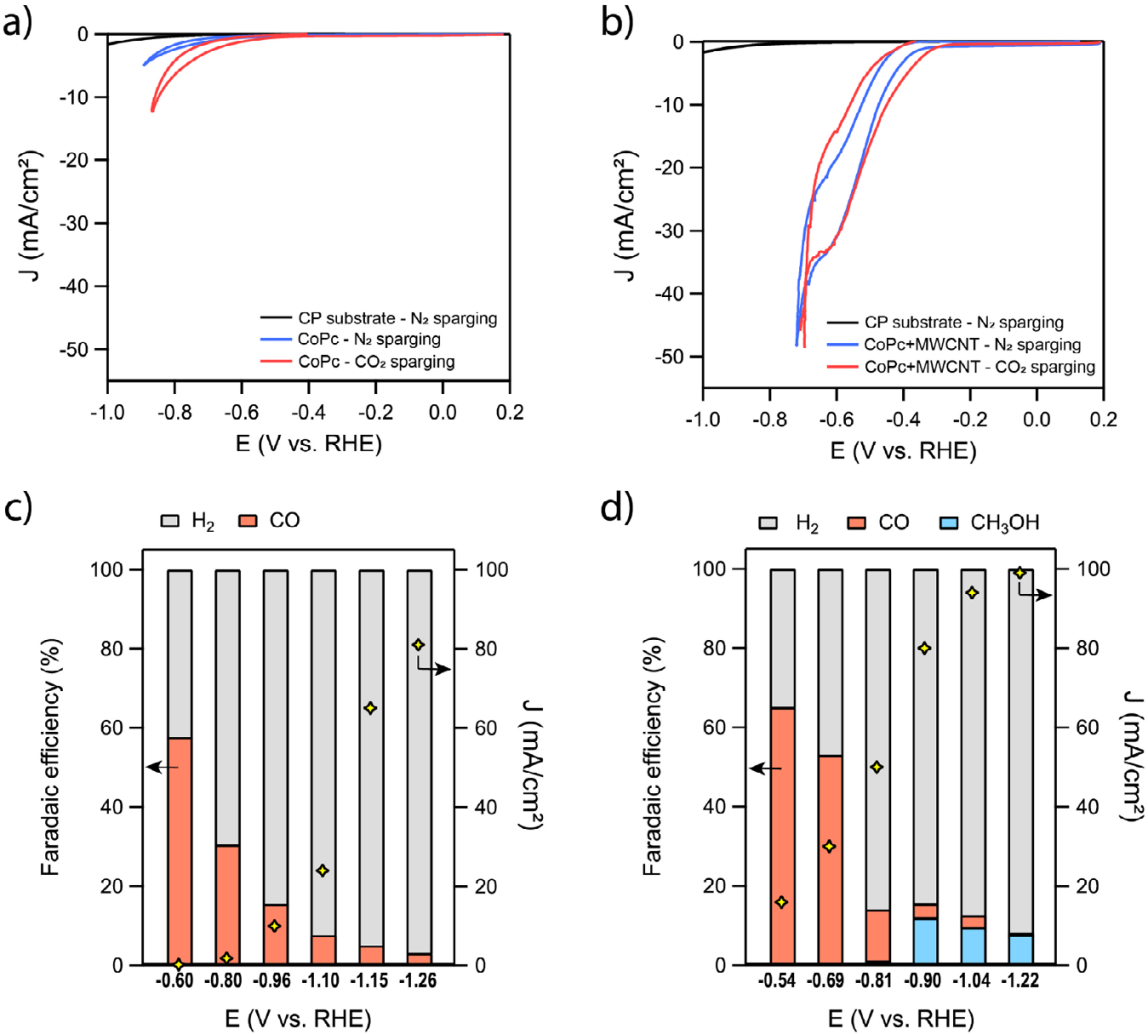
Cyclic voltammograms acquired at 20 mV/s in 0.5 M ${\mathrm{KHCO}}_{3}$ in the absence (blue trace) and presence (red trace) of ${\mathrm{CO}}_{2}$ sparging at 50 mL/min for CoPc a) and CoPc+MWCNT b) electrocatalysts coated on a CP substrate. Faradaic efficiencies measured for $H_{2}$, CO and methanol after 2 h of polarization at different potentials for CoPc c) and CoPc+MWCNT d) electrocatalysts coated on a CP substrate. The corresponding average current densities are indicated by yellow markers and referenced to the right Y‐axis. Electrochemical potentials have been corrected for the ohmic drop in all cases.

CoPc/CP and CoPc+MWCNT/CP samples were subjected to electrochemical testing in a two‐compartment H‐cell containing a 0.5 M ${\mathrm{KHCO}}_{3}$ electrolyte. Each material was first evaluated via CV under both ${\mathrm{CO}}_{2}$ ‐free and ${\mathrm{CO}}_{2}$ ‐saturated conditions (see Figure 6a and b).

In the case of CoPc deposited directly onto carbon paper, the CV in the absence of ${\mathrm{CO}}_{2}$ was featureless at potentials anodic to –0.4 V vs. the reversible hydrogen electrode (RHE), with a cathodic current observed at more negative potentials, attributed to the hydrogen evolution reaction (HER). We note that, based on a CV performed inside a dry organic electrolyte (Figure S11) and previous reports [65], we expect the active species for HER, and CO2RR, to be ${\mathrm{CoPc}}^{2-}$ (where one charge lies on the Co (I) center and the other is delocalised on the macrocyclic ligand). Upon ${\mathrm{CO}}_{2}$ sparging, a modest increase in cathodic current suggested electrocatalytic activity toward ${\mathrm{CO}}_{2}$ reduction. Chronoamperometry (CA) measurements carried out for 2 h at potentials between –0.6 and –1.6 V vs. RHE (Figure S12) confirmed the formation of CO as the sole carbon‐containing product, with a maximum Faradaic efficiency of 65%. Molecular hydrogen dominated the product distribution at more cathodic potentials, and the overall current densities remained low (–0.5 to –3.0 mA ${\mathrm{cm}}^{-2}$ in the –0.6 to –0.8 vs RHE range), consistent with limited ${\mathrm{CO}}_{2}\mathrm{RR}$ activity under these conditions. The FE for CO production, while obtained under non‐optimized conditions, represents a solid performance for a molecular catalyst operating without nanostructured supports.

The CoPc+MWCNT/CP electrode exhibited a different electrochemical signature. In the absence of ${\mathrm{CO}}_{2}$, the CV revealed a distinct redox couple located at ca. ‐0.6 V vs RHE ascribed to the ${\mathrm{CoPc}}^{-}$/${\mathrm{CoPc}}^{2-}$ transition, superimposed on the HER region. This redox signal indicates enhanced electrochemical accessibility of the molecular catalyst, likely due to improved dispersion and interfacial contact with the nanotube support, thereby increasing current densities. Upon ${\mathrm{CO}}_{2}$ introduction, the CV profile remained largely unchanged, though the possibility of ${\mathrm{CO}}_{2}$ reduction occurring concurrently with HER cannot be excluded based on voltammetry alone. Subsequent CA experiments under identical conditions revealed CO as a major product at moderate potentials, with methanol detected at more negative potentials. 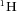 NMR analysis confirmed this species as the sole liquid product, as displayed in Figure S14. A maximum Faradaic efficiency of 12% for methanol was observed at –0.9 V vs. RHE, in agreement with previously reported values for CoPc‐based systems [66]. Still, it is important to note that the CA measurements presented in Figure S12 reveal a stable current density over the course of 2 h, suggesting no drastic degradation (e.g. poisoning, mechanical detachment, demetallation...) of the electrocatalyst. This is further supported by SEM analysis of the electrode after testing at ‐1.0 V vs. RHE for 2 h, where no significant change in morphology was observed compared to the fresh sample (Figure S13).

Still, after 2 h it is likely that the selectivity toward methanol has started to drop due to the progressive hydrogenation of the macrocyclic ligand, as reported previously [35, 39, 67]. According to previous theoretical and experimental work performed on the system, the conversion of CO_2_ to methanol proceeds through the generation of a CO* intermediate after a 2‐electron reduction step. The interaction of the CoPc with the MWCNT is thought to modulate the energy of the CoPc‐CO* intermediate so that the carbonyl can be more easily reduced to methanol rather than desorbing readily to generate CO(g), as is observed on unsupported CoPc. The hydrogenation of the CoPc will however affect the energies of the transition states associated with CO and methanol production and revert the selectivity back to the former over time. [34, 68, 69]

## 4 Conclusion

We have developed an efficient and environmentally conscious synthetic procedure for cobalt phthalocyanine (CoPc), employing low‐impact solvents and simplified purification to minimize waste and resource consumption. Through systematic optimization of reaction conditions, the protocol achieved an 85% yield and an E‐factor of 18.6, positioning it as a relevant contribution to ongoing efforts in sustainable lab‐scale phthalocyanine synthesis. These results demonstrate that high‐yield synthesis can be achieved without compromising environmental responsibility, reinforcing the potential of this method as a sustainable alternative to conventional routes. CoPc was subsequently validated as an active molecular electrocatalyst for the electrochemical reduction of ${\mathrm{CO}}_{2}$. Comprehensive morphological and spectroscopic analysis confirmed that the catalyst maintains its integrity, while the support architecture modulates the dispersion and accessibility of its active sites. When supported on carbon nanotubes, the green CoPc exhibits structural and chemical characteristics comparable to those of conventionally synthesized analogues, achieving methanol production with a Faradaic efficiency of up to 12%, consistent with literature values. These findings validate the compatibility of green‐synthesized molecular catalysts with existing ${\mathrm{CO}}_{2}\mathrm{RR}$ technologies, offering a more environmentally conscious route to methanol production without sacrificing catalytic performance. This work highlights the importance of integrating green chemistry principles early in catalyst development. As electrochemical ${\mathrm{CO}}_{2}$ reduction technologies move toward commercialization, such sustainable approaches will be essential to reduce environmental impact across the entire value chain. Future efforts will focus on optimizing catalyst integration and scaling performance in device‐relevant configurations.

## Conflicts of Interest

The authors declare no conflict of interest.

## Supporting information


**Supporting File 1**: chem70461‐sup‐0001‐SuppMat.pdf.
