# Supplementary material for: Eco‐Friendly Synthesis of Cobalt Phthalocyanine for High‐Efficiency CO2 Electroreduction
Source: Chemistry. 2025 Nov 26;32(29):e02264. doi: 10.1002/chem.202502264 (PMC13451542; doi:10.1002/chem.202502264)
Supplement: Supplementary file 1 [file CHEM-32-e02264-s001.pdf]

ORIGINAL ARTICLE

Supporting Information

Eco-Friendly Synthesis of Cobalt Phthalocyanine for High-Efficiency CO_2 Electroreduction

Gloria Zanotti[1] | Federica Palmeri[1,2] | Venanzio Raglione[3] | Abdolhamid Khodadadi[3] | Camille Tabaries[4] | Jerome Capitolis[4] | Mathieu Prevot[4] | Laurent Piccolo[4] | Mahmoud Zendehdel[5,6] | Roberto Flammini[3] | Giorgio Contini[3]

[1]Istituto di Struttura della Materia-CNR (ISM-CNR), Strada provinciale 35d/9, 00010 Montelibretti, Italy

[2]Department of Chemistry, La Sapienza University of Rome, Piazzale Aldo Moro, 5, 00185 Roma, Italy

[3]Istituto di Struttura della Materia-CNR (ISM-CNR), Via del Fosso del Cavaliere, 00133 Roma, Italy

[4]Université Claude Bernard Lyon 1, CNRS, Institut de Recherche sur la catalyse et l'environnement (IRCELYON, UMR 5256), 2 Av. A. Einstein, F-69626 Villeurbanne Cedex, France

[5]Iritaly Trading Company S.r.l., Via Volturno 58, 00185 Roma, Italy

[6]Dept. Electronics Engineering, Univ. of Rome "Tor Vergata", via del Politecnico 1, 00133 Roma, Italy

Correspondence
Giorgio Contini PhD, Roberto Flammini PhD, Istituto di Struttura della Materia-CNR (ISM-CNR), Via del Fosso del Cavaliere, 00133 Roma, Italy
Email: giorgio.contini@cnr.it; roberto.flammini@cnr.it

Funding information
NEXTCCUS project (ID: 327327), which is funded through the ACT program (Accelerating CCS Technologies, Horizon2020; Project No. 691712).

XPS wide scans, C1s and F1s core level spectra, along with a SEM side view image of the substrate are provided. The ^{1}H NMR spectra of phthalonitrile, phthalimide, and CoPc in DMSO-d_6, are also reported, along with the spectrum of a defined mixture of them in the same deuterated solvent. Complementary spectroscopic and mass spectrometric data include the electronic absorption spectrum of CoPc in tetrahydrofuran (THF) solution, the FTIR spectrum of CoPc powder, and the atmospheric pressure MALDI (AP-MALDI) mass spectrum. Electrochemical characterizations comprise the cathodic scan of a CoPc solution in DMF and the chronoamperometric curves recorded for the two samples. SEM images of CoPc/CP and CoPc+MWCNT/CP, freshly prepared and after operation, are also presented. Finally, the ^{1}H NMR spectrum of an electrolyte sample collected during CO_2 electroreduction is shown. Complete details are provided on the calculation of the environmental factor (E-factor).

Supporting Information

In Fig.S1 panel (a), the XPS wide-scan spectra of different samples are shown. As expected, the C1s peak is present in all samples. The fluorine peak originates from the CP (see main text), and its intensity is reduced in the CoPc+MWCNT/CP and MWCNT/CP samples due to coating thickness. Oxygen is present in all samples except for CP. In the CoPc crystallite sample (CoPc/CP), the intense Co2p peaks indicate a thick coating of CoPc molecules. Correspondingly, in the CoPc+MWCNT/CP sample, the Co peak shows a reduced intensity due to the dispersed phase of spray-coated CoPc on the MWCNTs. Similarly, nitrogen, which is found exclusively in CoPc, follows the same trend as cobalt. Additionally, we observed Auger peaks associated with fluorine, labeled as F(KLL). The core-level spectra of the two main elements present in CP, C1s and F1s, are displayed in Fig.S1 panels (b) and (c), respectively (see Ref. [1] in this Supplementary Information).

In Fig. S2 and S3, side-view SEM images of both samples are shown, illustrating the composition of the substrates. The upper layer represents the CoPc mixture coating, while the lower part corresponds to the carbon paper.

Top view, SEM images of the sample containing MWCNTs, with and without CoPc, are displayed in Fig. S4.

Fig. S5, S6, S7 and S8 show the ^{1}H NMR characterization of the target compound and potential byproducts in DMSO-d_6, as well as the spectrum of a mixture prepared in defined proportions.

In Fig. S9 the electronic absorption spectrum of CoPc in tetrahydrofuran (THF) solution is shown.

In Fig. S10, the FTIR spectrum of CoPc powder is shown. As expected, the main peak contributions appear below 1700 cm^{-1} and are primarily due to the vibrational modes of the macrocycle.

Fig. S11 shows the cathodic scan of a CoPc solution in dimethylformamide (DMF).

In Fig. S12 chronoamperometric curves relative to the two samples are displayed.

Fig. S13 shows the SEM images of CoPc/CP and CoPc+MWCNT/CP, freshly prepared and after operation, illustrating the morphological changes occurring over time.

Fig. S14 shows the ^{1}H spectrum of an electrolyte sample taken during the electroreduction ofCO_2, used for methanol detection and quantification with DMSO as the internal standard.

Fig. S15 shows the AP-MALDI spectrum of CoPc, recorded in positive-ion mode, with the compound detected as the radical cation $[\mathrm{C_{32}H_{16}N_8Co}]^{\bullet+}$.

The Environmental factor (E-factor) is a mass-based metric defined as the amount of waste generated to produce one gram of product. Water and recovered solvents or reactants are not included in the calculation. It is calculated as:

$$\text{E-factor} = \frac{\text{reagents + solvents - product}}{\text{product}}.$$

For CoPc synthesis, the E-factor is calculated as 18.6.

For the cost estimation of CoPc synthesis, Merck and CarloErba were selected as suppliers for reagents and solvents. The largest available batches with sufficient purity were chosen from their online catalogs. The product numbers for each chemical are listed in Tab.s S1 and S2 for clarity. Prices were rounded to the nearest cent. Costs related to energy, facility maintenance, personnel, taxes, and other expenses were not included in this estimation. Prices were verified as of June 30th, 2025.

references

[1] Okpalugo TIT, Papakonstantinou P, Murphy H, McLaughlin J, Brown NMD. High resolution XPS characterization of chemical functionalised MWCNTs and SWCNTs. Carbon 2005;43(1):153–161. https://www.sciencedirect.com/science/article/pii/S0008622304005317.

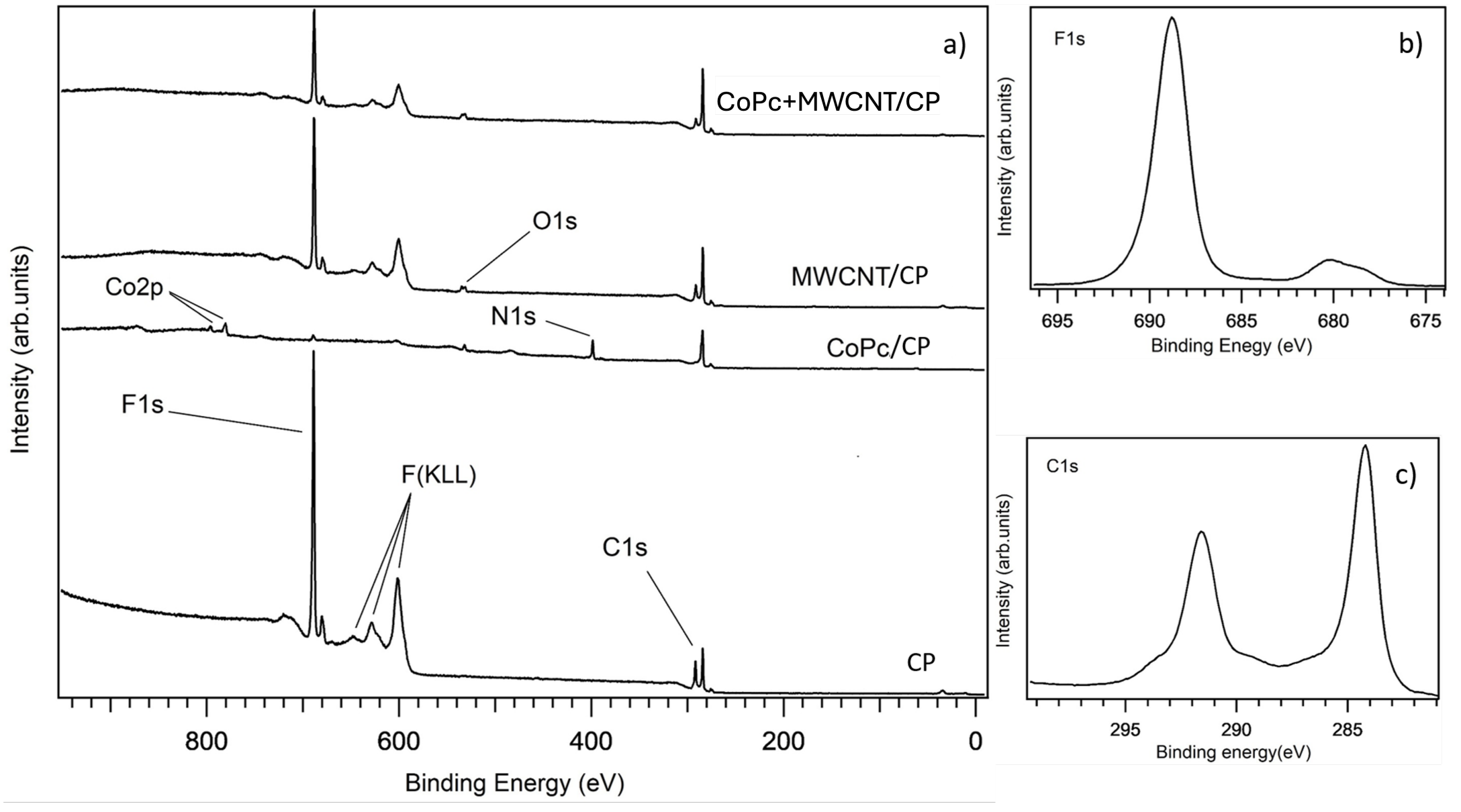

FIG. S1 (a) Comparison of the wide XPS spectra recorded for CoPc+MWCNT/CP, MWCNT/CP, CoPc/CP, and CP (from top to bottom). (b, c) C1s and F1s core-level spectra recorded on CP, respectively.

TAB. S1 Cost estimation for obtaining 1.0 g of CoPc at 85% yield, assuming linear scalability of the selected reaction conditions. The quantities were calculated by proportionally scaling the experimental reagent amounts based on a reaction that yielded 0.941 g of CoPc. Unit costs are listed per gram of each reagent.

Chemical	Experimental amount (g)	Scaled amount for 1.0 g of product	Cost/g (EUR)
Phthalonitrile	1.0	1.1	0.13
Glycerol	3.0	3.2	0.07
Anisole	0.3	0.32	0.01
$Co(OAc)_2\ 4H_2O$	0.51	0.54	0.12
DBU	0.016	0.017	0.00
HCl 1M	1.0	1.1	0.01
Methanol	12.7	13.5	0.14
**Total estimated cost per gram of CoPc**			**0.48**

TAB. S2 Reference prices used to estimate the cost of CoPc synthesis. Densities are provided for all liquid compounds and were used to calculate batch sizes for those available in litre-based quantities (i.e., sold by volume rather than by weight).

Name	Product No.	Supplier	Density (g/mL)	Size	Price (EUR)	Price/g (EUR)
Phthalonitrile	LA150710B	Carlo Erba	/	1.0 Kg	119.91	0.12
Glycerol	8187099025	Merck	1.26	25.0 L	679.00	0.02
Anisole	8014529025	Merck	0.995	25.0 L	866.00	0.03
$Co(OAc)_2$ $4H_2O$	208396-1KG	Merck	/	1.0 kg	224.00	0.22
DBU	8032829050	Merck	1.02	50.0 kg	3030.00	0.06
HCl 1M	110165902	Merck	1.02	25.0 L	312.00	0.01
Methanol	8222839180	Merck	0.792	180.0 L	1800.00	0.01
CoPc β-form, Purity 97%	307696-50G	Merck	/	50.0 g	691.00	13.82
CoPc, Purity >93%	P0887	TCI Chemicals	/	25.0 g	181.00	7.24
CoPc, Purity >95%	Catalog No. 067	Porphychem	/	5.0 g	40.00	8.00
CoPc	L04149614	Carlo Erba	/	25.0 g	522.71	20.91

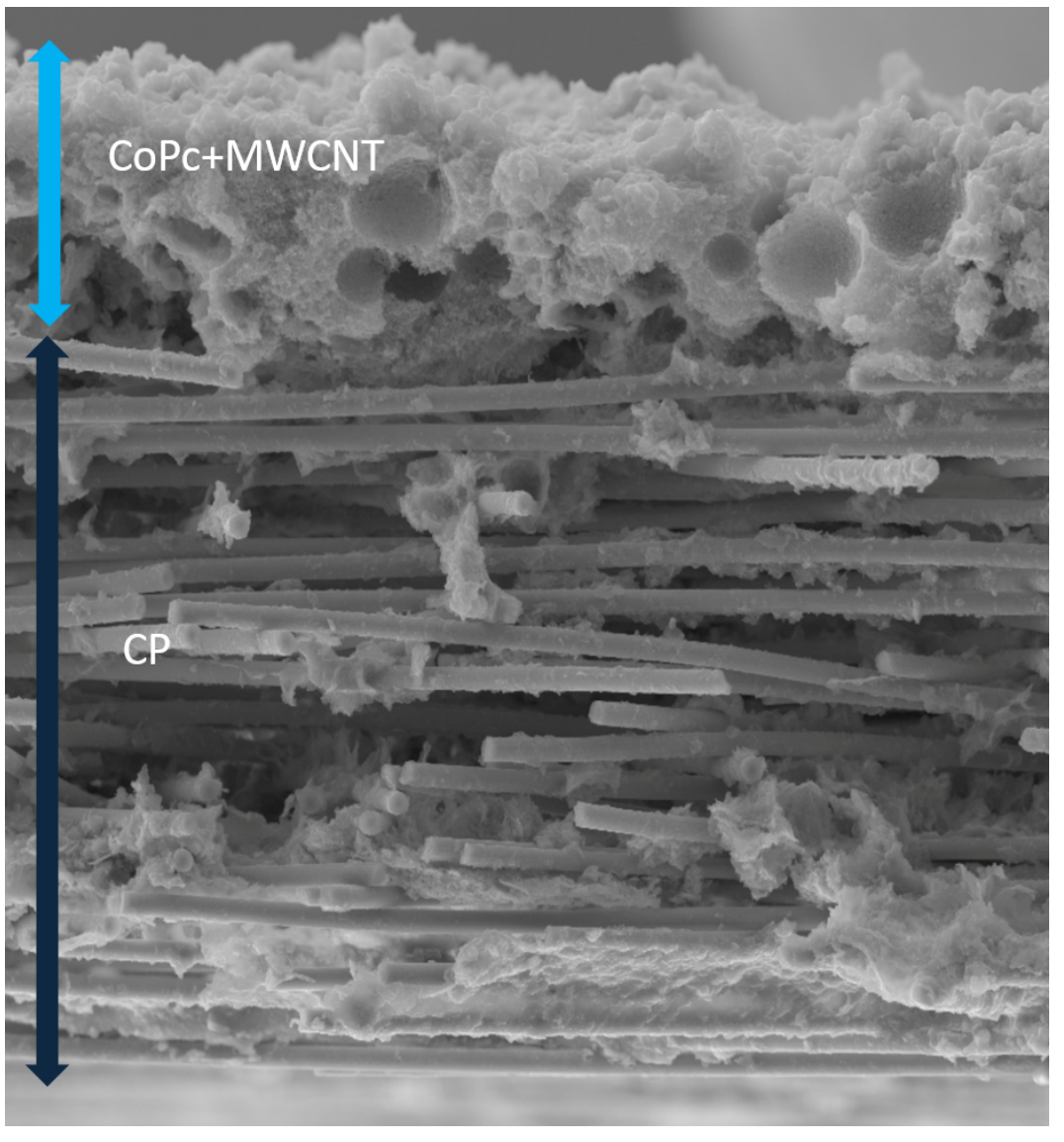

FIG. S2 SEM side-view image of the CoPc+MWCNT/CP substrate. 1kX magnification.

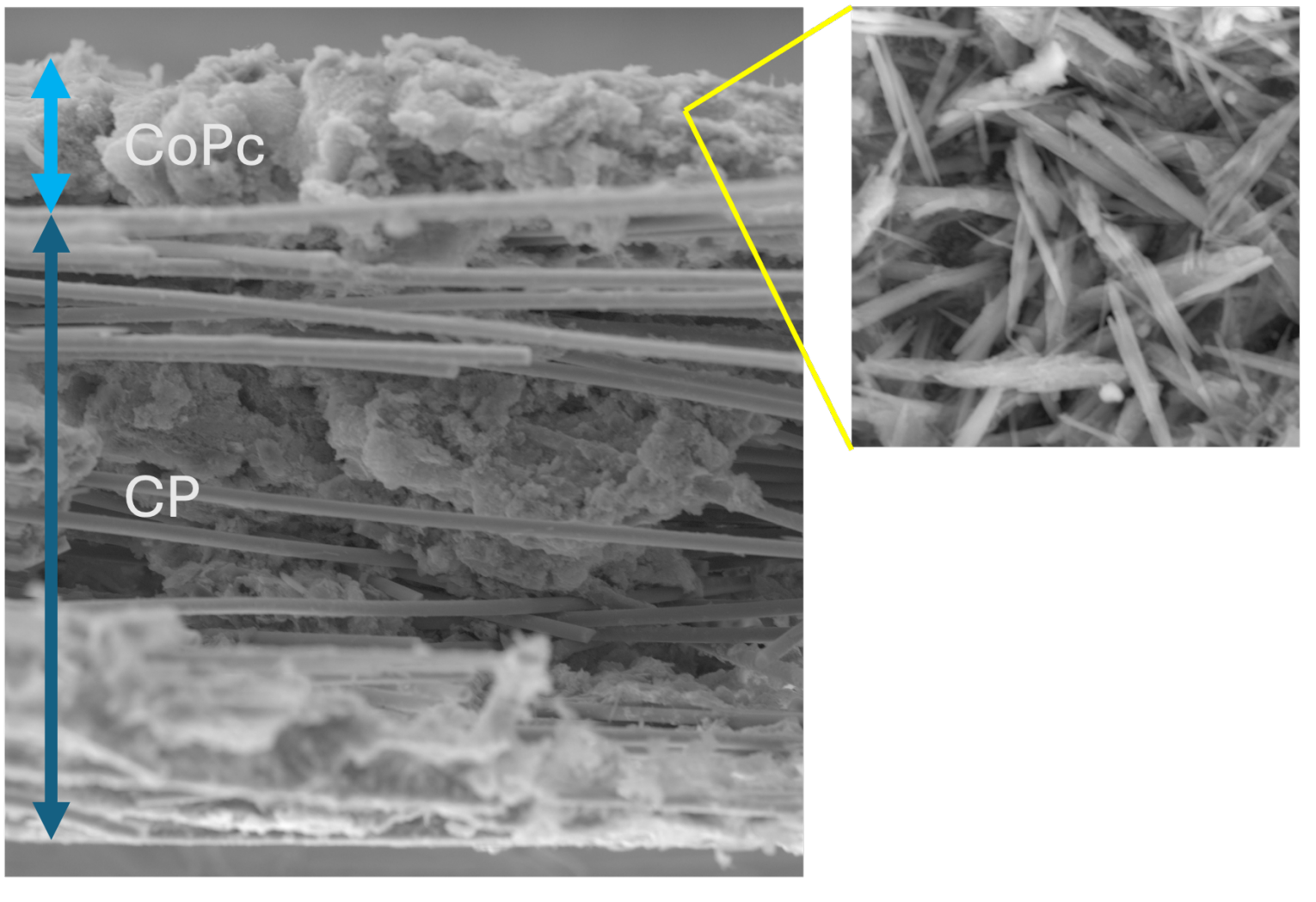

FIG. S3 SEM side-view image of the CoPc/CP substrate, 1kX magnification. In the inset, top view of the CoPc crystallites, 10kX magnification.

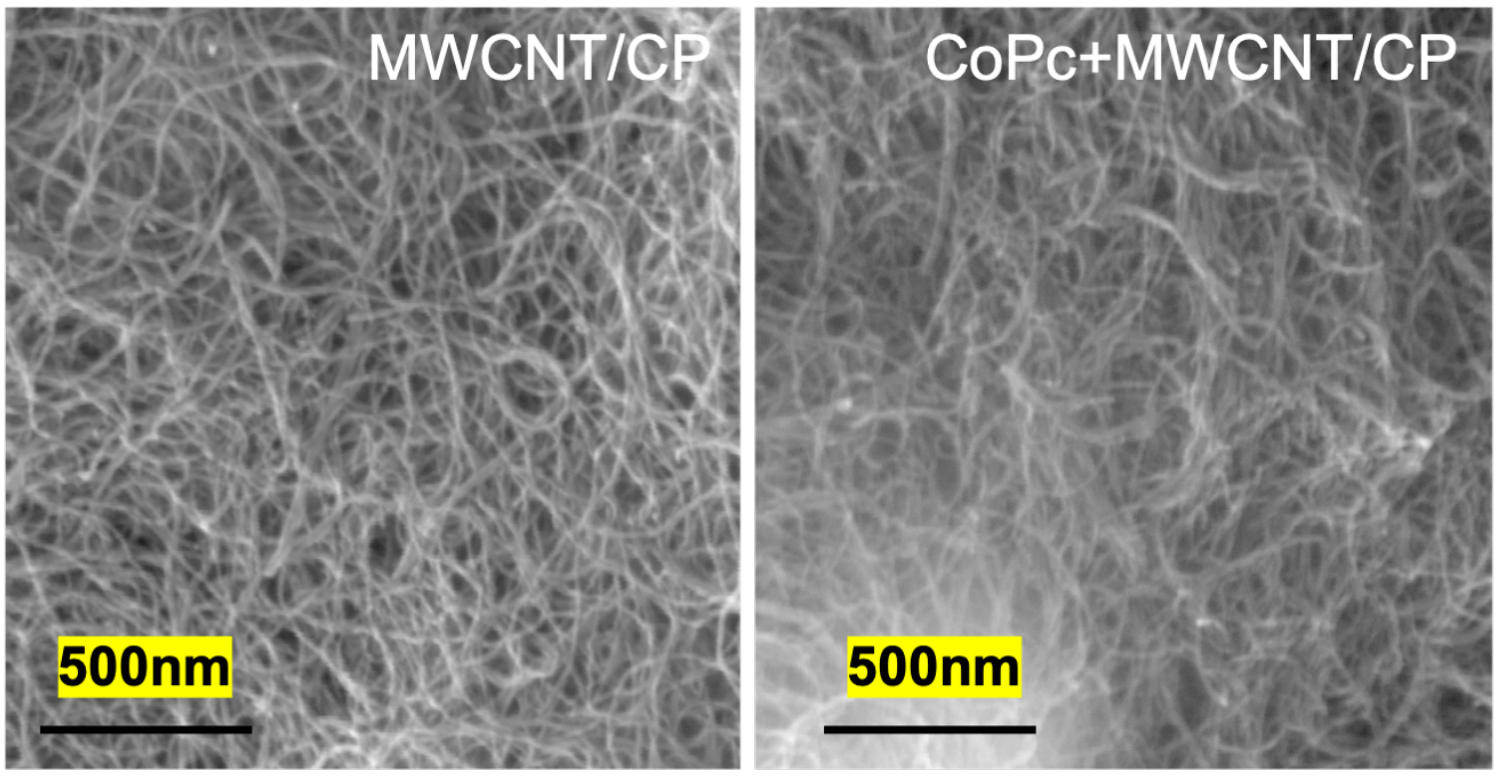

FIG. S4 SEM images of MWCNT-containing samples without (left panel) and with (right panel) CoPc at 20kX magnification.

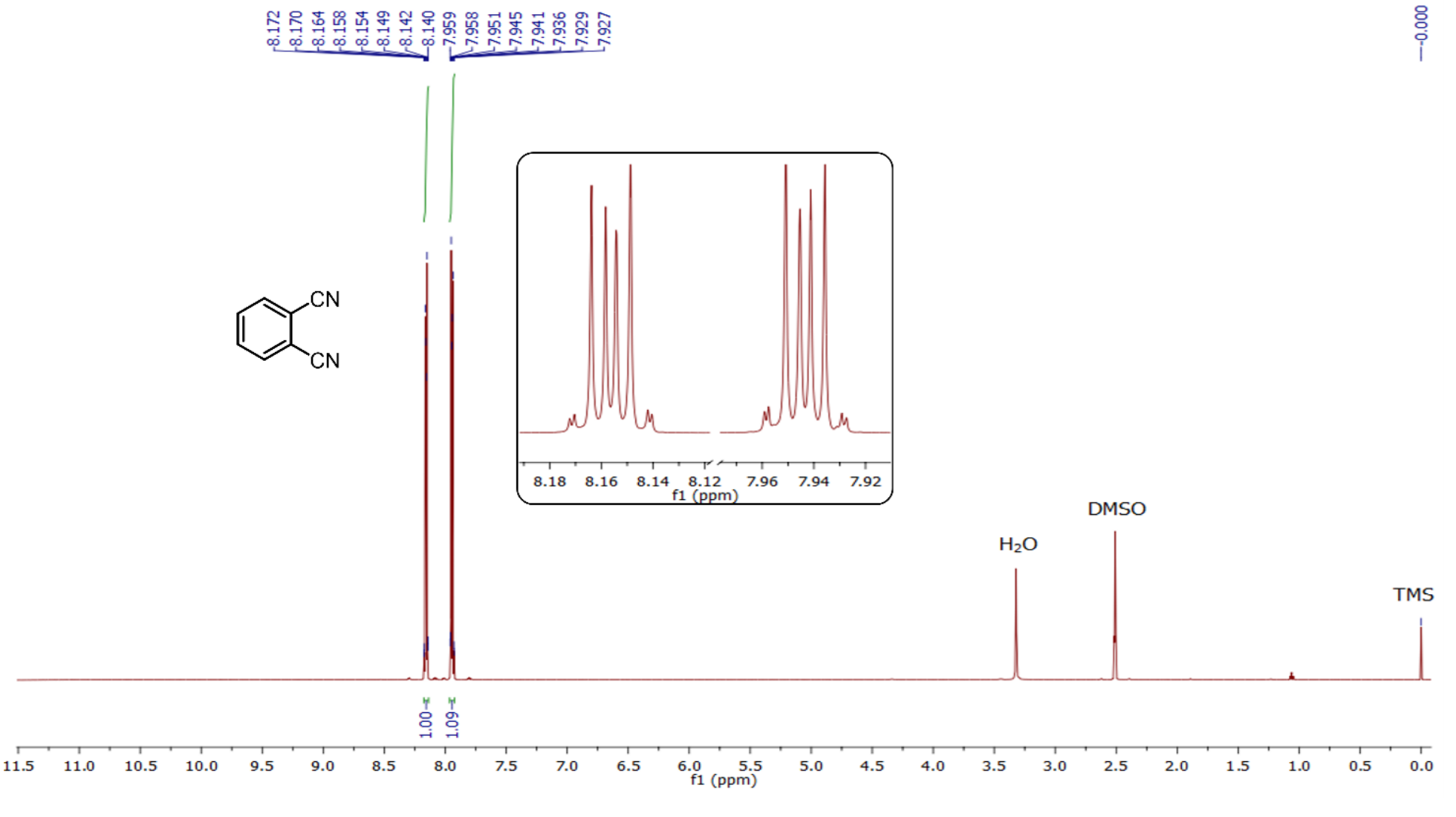

FIG. S5 ^{1}H NMR spectrum of phthalonitrile in DMSO-d_6.

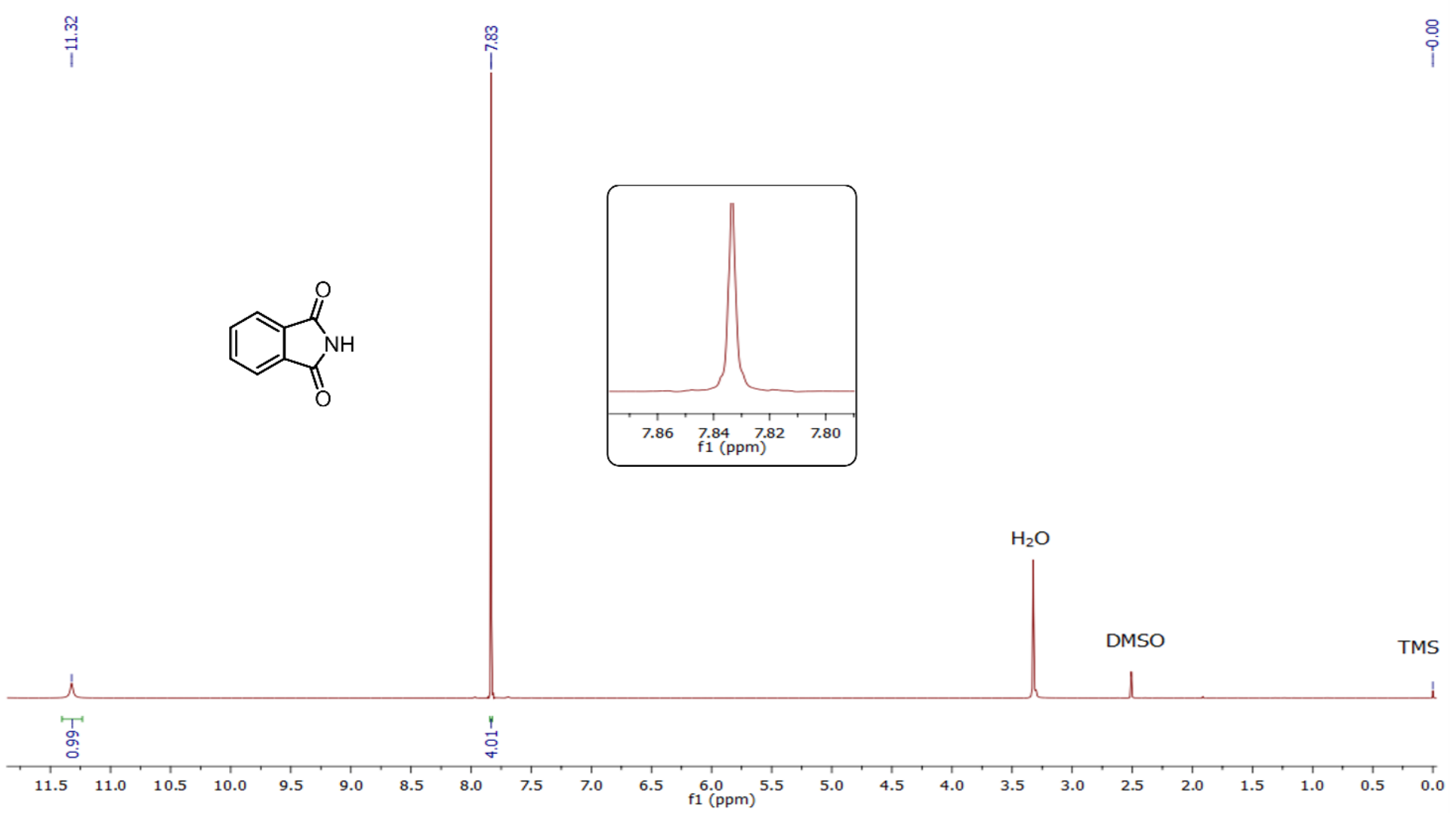

FIG. S6 ^{1}H NMR spectrum of phthalimide in DMSO-d_6.

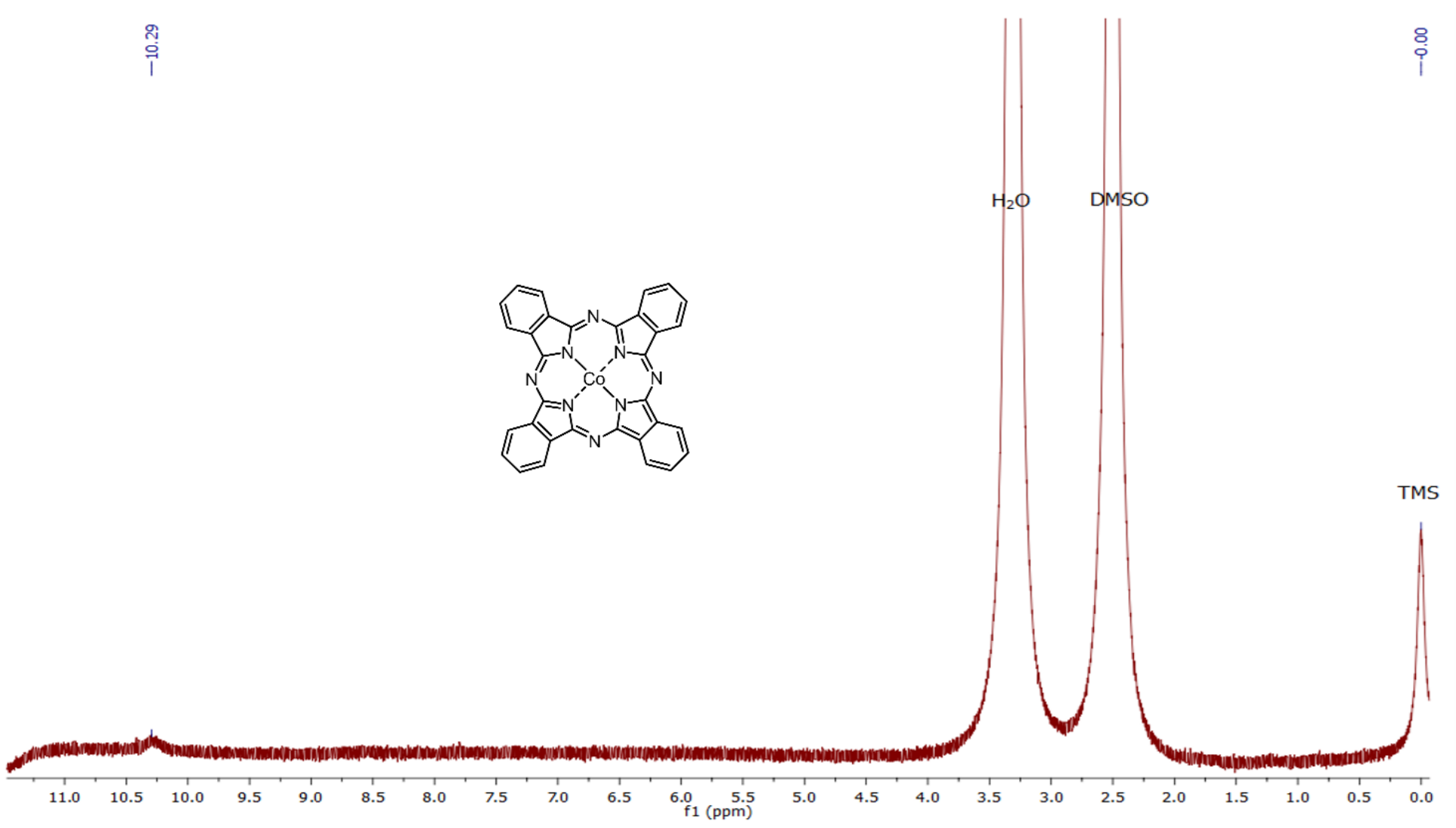

FIG. S7 ^{1}H NMR spectrum of cobalt phthalocyanine (1 mg) in DMSO-d_6. The weak, broad signal at 10.29 ppm is tentatively attributed to ring protons although unusually deshielded. If so, the two non-equivalent proton groups of the macrocycle appear to coalesce into a single resonance, as only one signal is detected instead of the expected two.

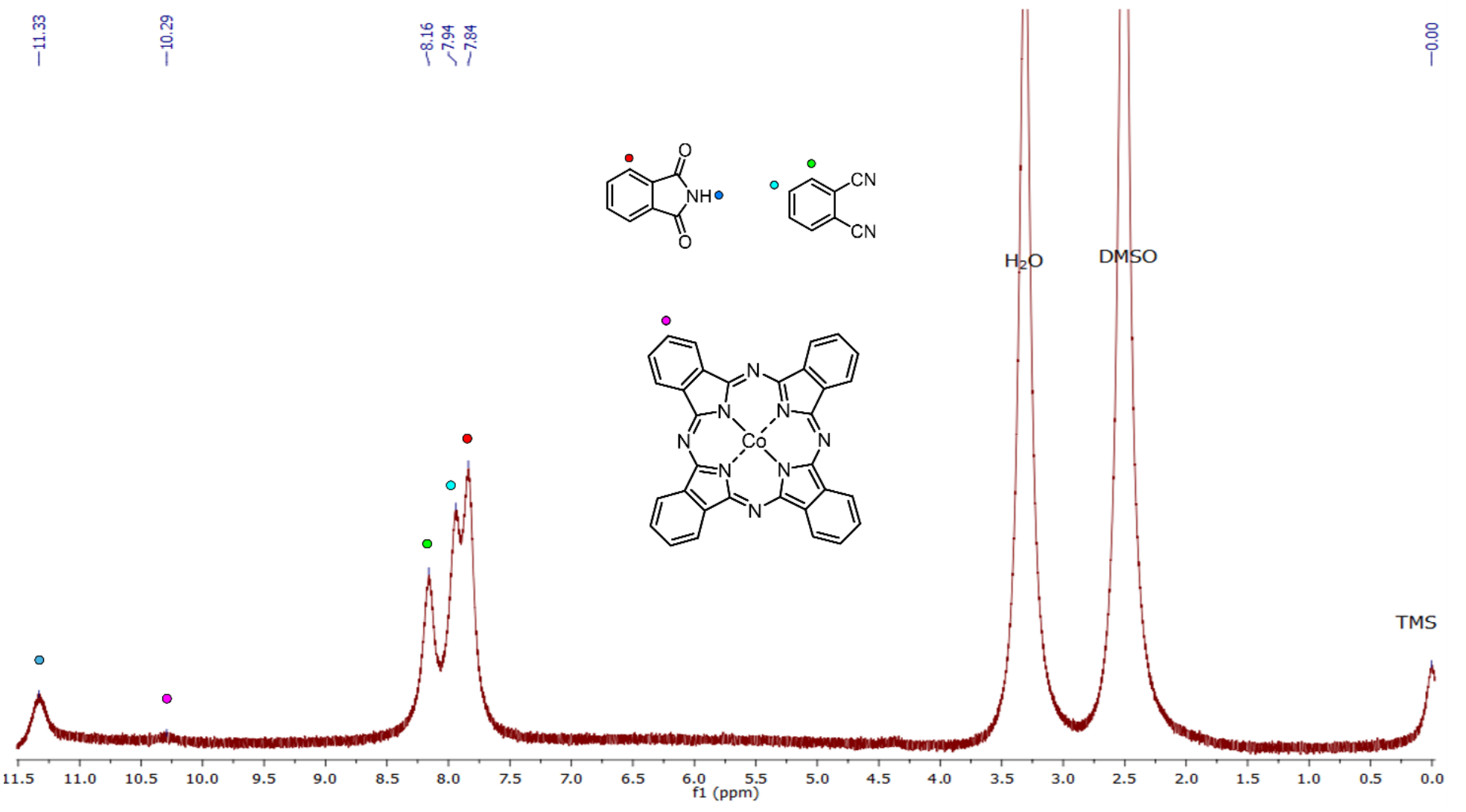

FIG. S8 ^{1}H NMR spectrum of a mixture of cobalt phthalocyanine (1 mg), phthalimide (0.7 mg, 4.7 μmol) and phthalonitrile (0.6 mg, 4.7 μmol) in DMSO-d_6. Although broadened and unresolved due to Co^{2+}paramagnetism, the chemical shifts of the aromatic compounds are consistent with those observed in their reference spectra.

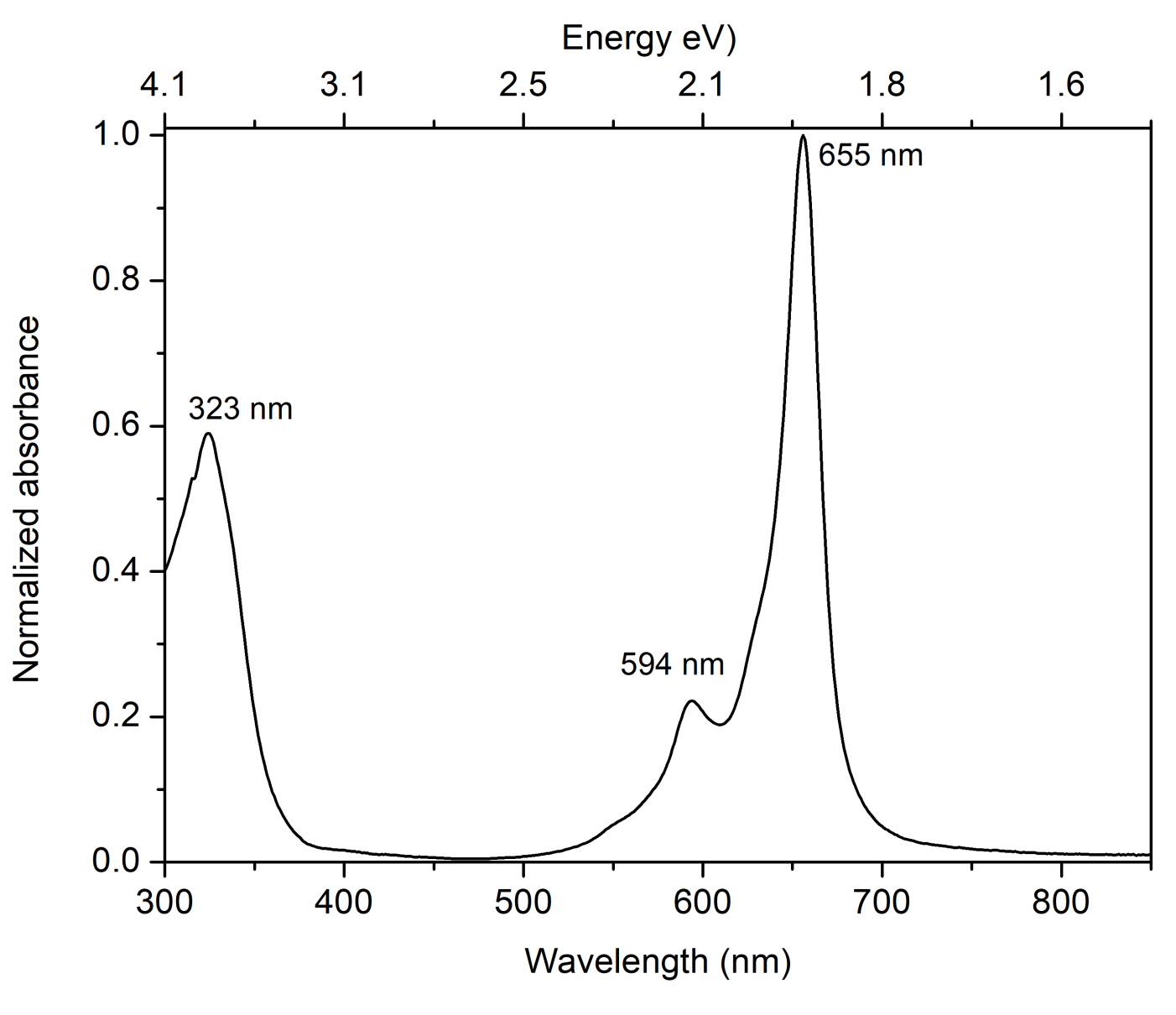

FIG. S9 UV-Vis spectrum of CoPc in THF solution.

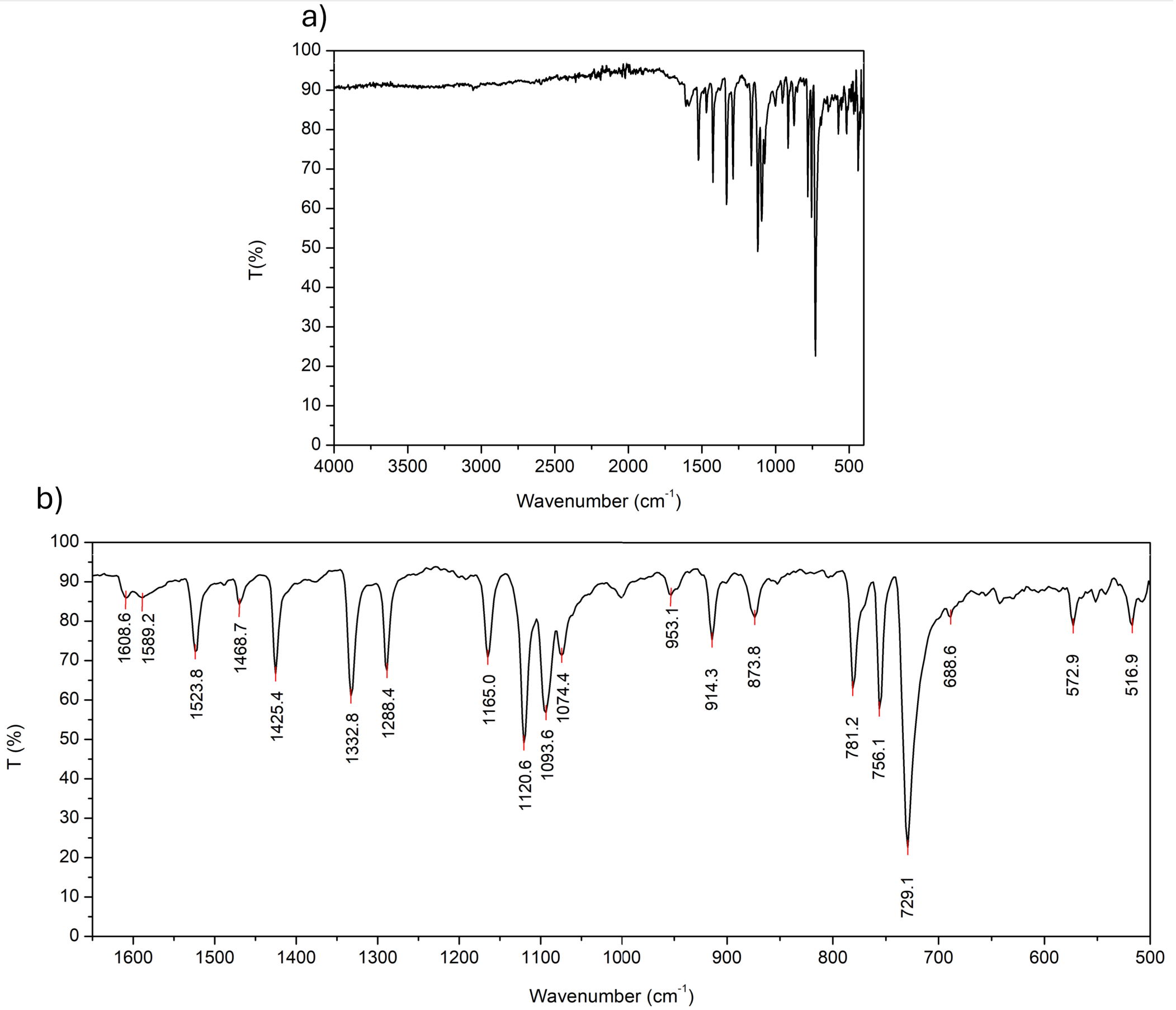

FIG. S10 IR spectrum of CoPc powder. a) Full spectrum recorded in the 4000–400 cm^{-1} range. b) Magnified view of the 1650–500 cm^{-1} region with peak assignments.

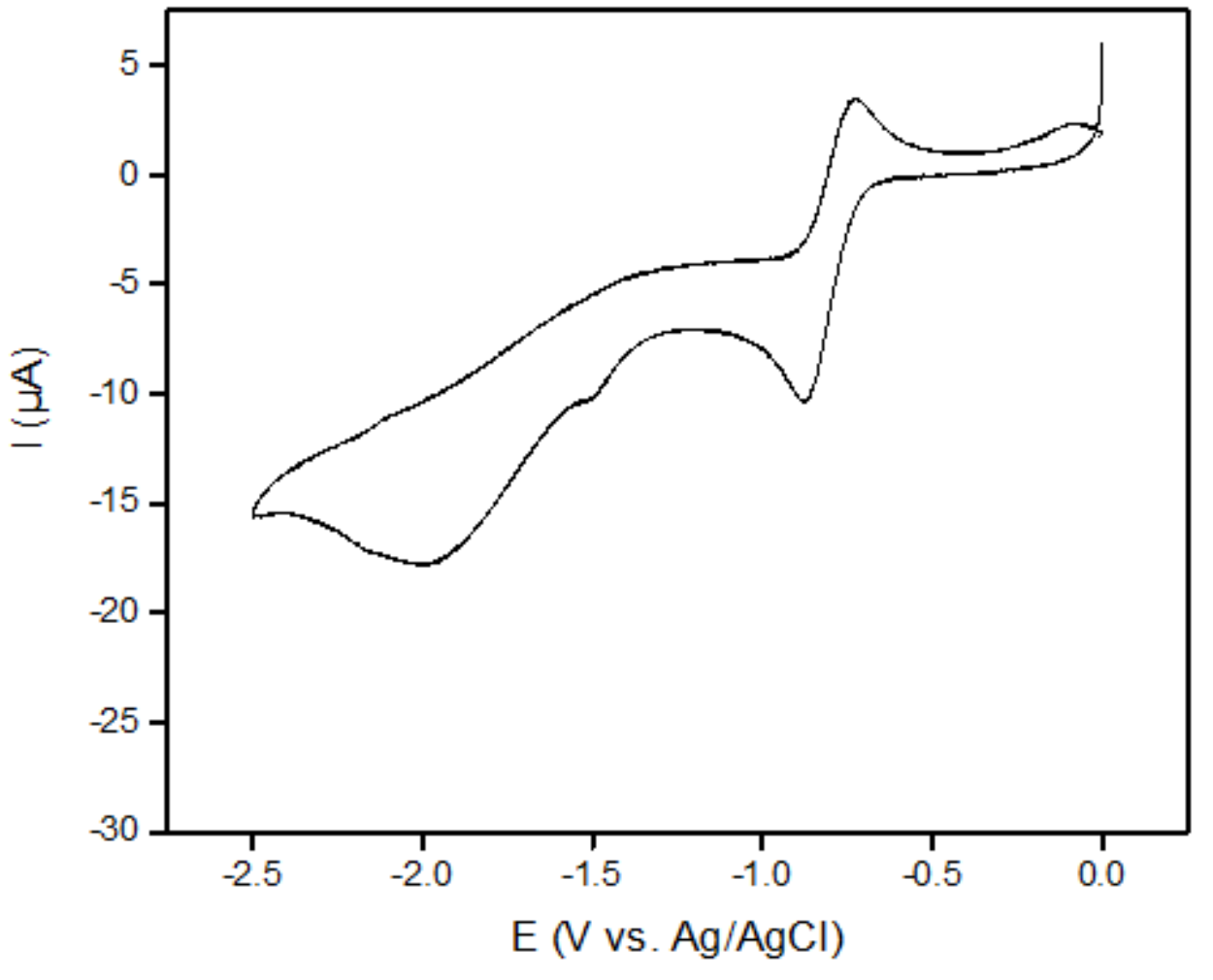

FIG. S11 Cyclic voltammetry performed at a glassy carbon electrode in an electrolyte of 0.1 M [TBAP][BF_6] in dry DMF. Counter electrode: platinum wire, reference electrode: Ag/AgCl/DMF. Scan rate: 20 mV/s. The waves at -0.7 V correspond to Co(II)/Co(I)-, while the $CoPc^{-}/CoPc^{2-}$ reduction wave is located at -1.5 V.

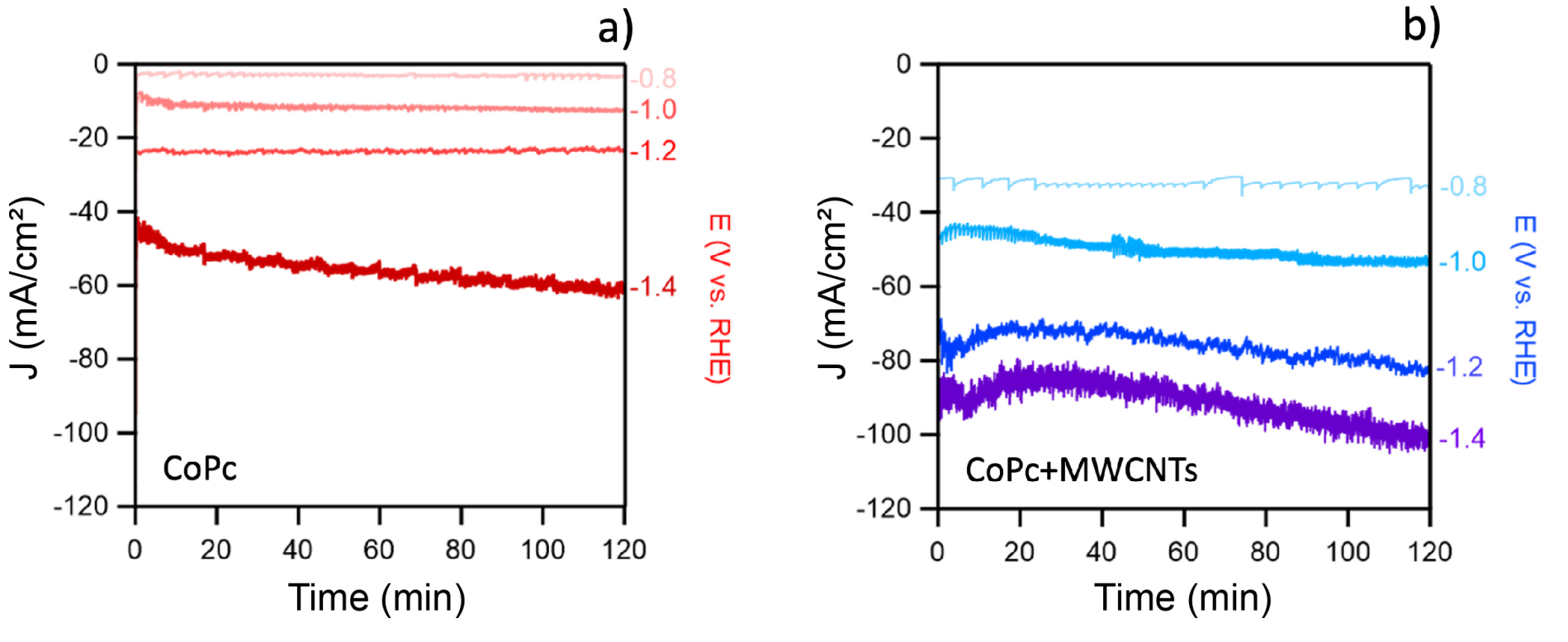

FIG. S12 Chronoamperometry performed over 2 hours using CoPc/CP and CoPc+MWCNT/CP electrodes at different applied potentials (indicated on the right side of each figure), panels (a) and (b), respectively.

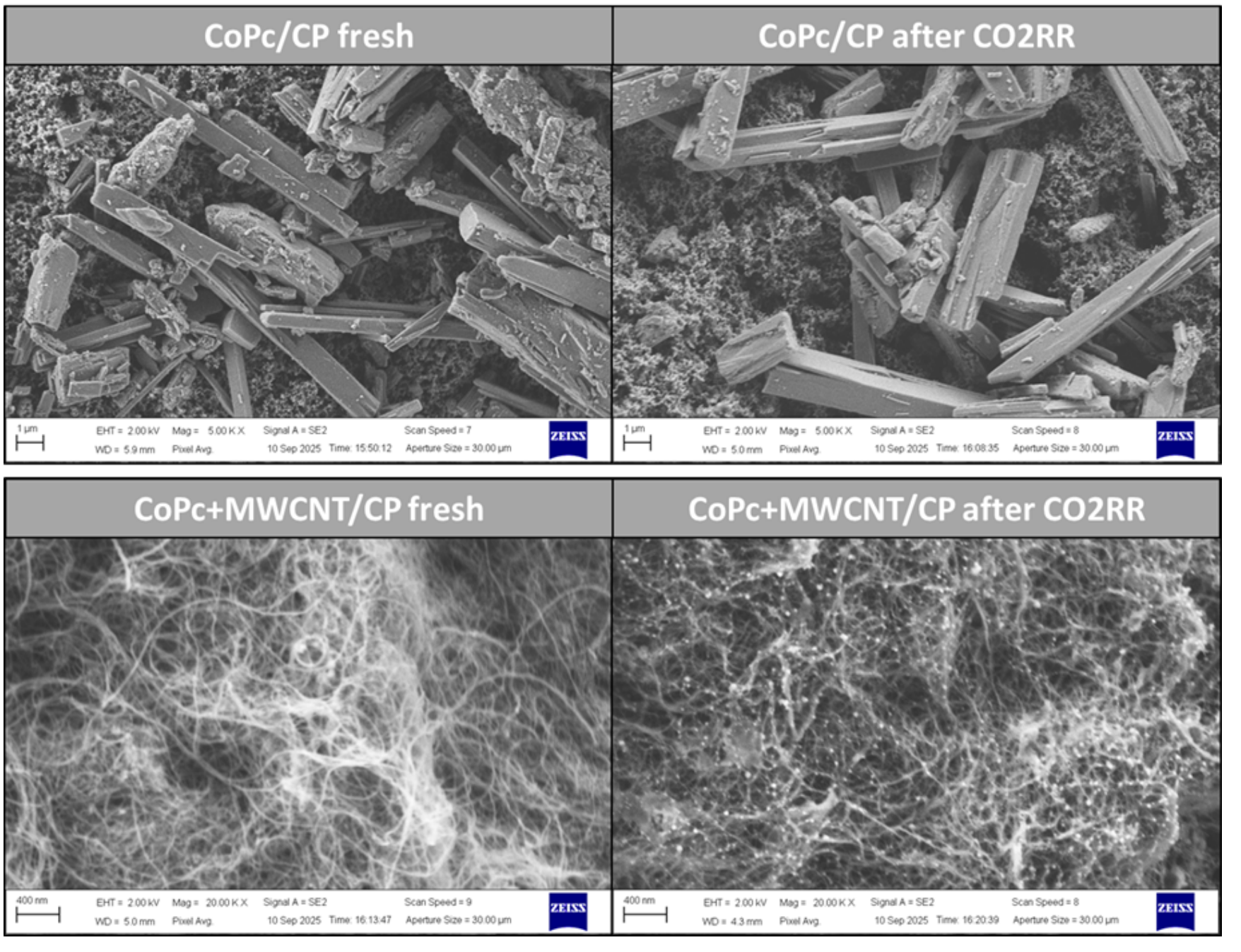

FIG. S13 SEM microscope picture of CoPc/CP and CoPc+MWCNT/CP freshly prepared and after one hour of operation at -1.0 V vs RHE for one hour in 0.5 M $KHCO_3$ under CO_2sparging at 50 mL/min

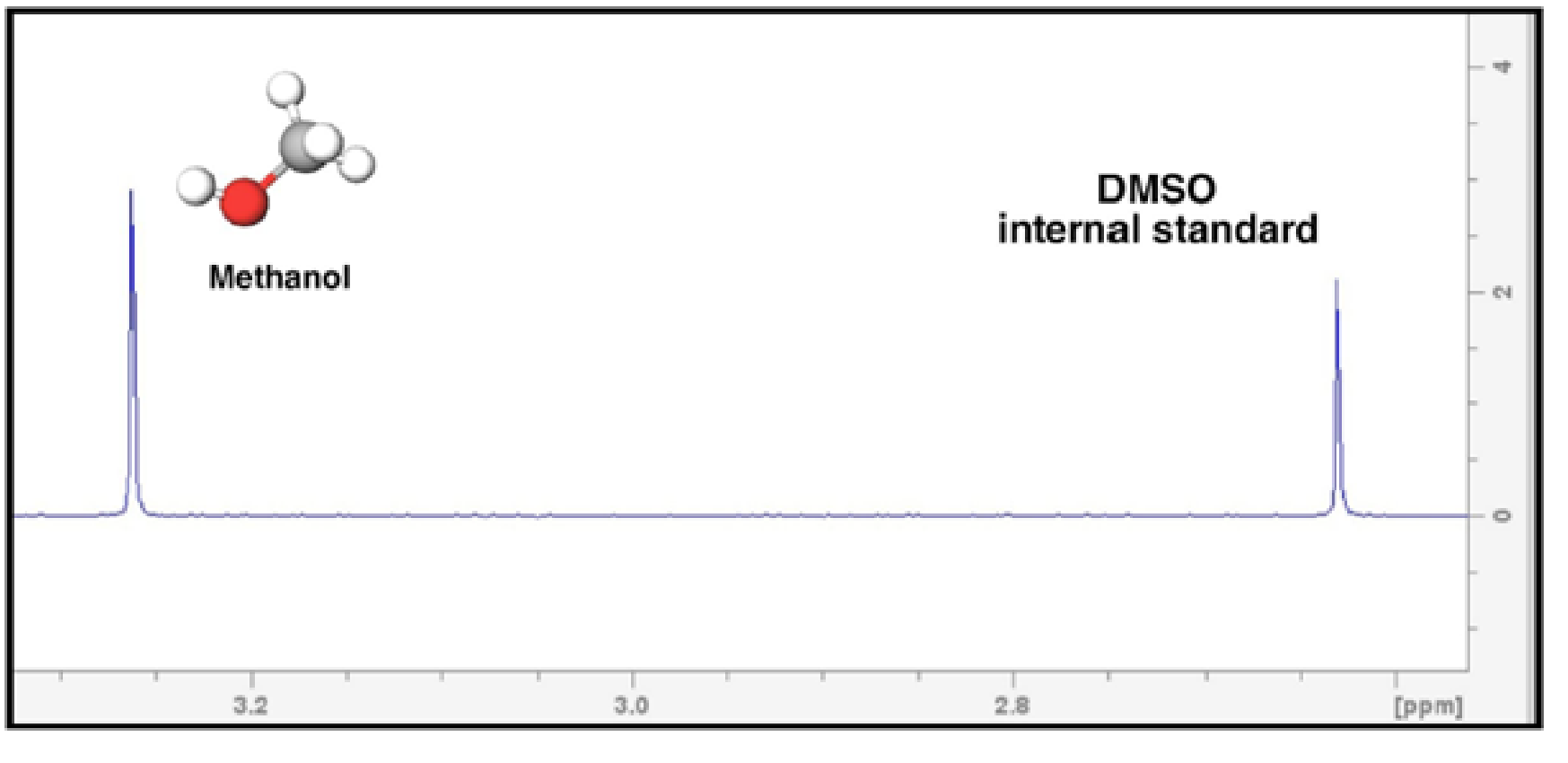

FIG. S14 Typical NMR spectrum of an electrolyte sample, used for methanol detection and quantification with a DMSO internal standard. The peak at 3.25 ppm corresponds to three protons of the methanol molecule, while the peak at 2.7 ppm corresponds to the six protons of the DMSO molecule.

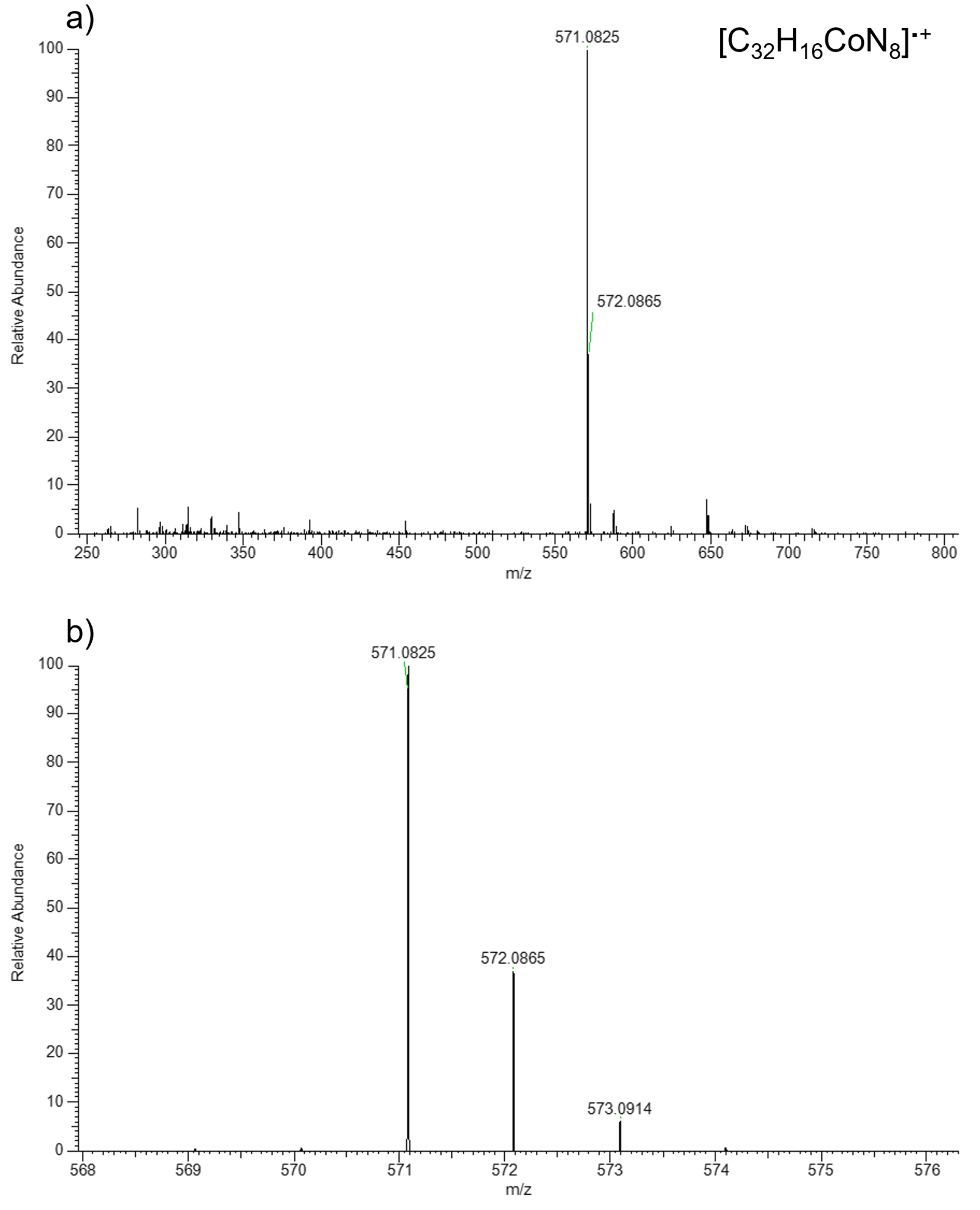

FIG. S15 a) AP-MALDI-(+) mass spectrum of CoPc in in the 250-800 m/z range; b) Magnification of 568-576 m/z region showing the isotopic pattern and compound attribution for the radical cation corresponding to $[C_{32}H_{16}N_8Co]^{\bullet+}$. Theoretical mass: 571.08244; Found: 571.0825 (error: 0.13 ppm).
